# Prolonged hematological toxicity in patients receiving BCMA/CD19 CAR-T-cell therapy for relapsed or refractory multiple myeloma

**DOI:** 10.3389/fimmu.2022.1019548

**Published:** 2022-10-18

**Authors:** Hujun Li, Lina Zhao, Zengtian Sun, Yue Yao, Li Li, Jiaojiao Wang, Tian Hua, Shengwei Ji, Shiyuan Wang, Hai Cheng, Ming Shi, Zhenyu Li, Lingyu Zeng, Qingyun Wu, Jianlin Qiao, Chong Chen, Junnian Zheng, Jiang Cao, Kailin Xu

**Affiliations:** ^1^ Department of Hematology, The Affiliated Hospital of Xuzhou Medical University, Xuzhou, China; ^2^ Department of Hematology, the First People’s Hospital of Lianyungang, Lianyungang, China; ^3^ The First Clinical Medical College, Xuzhou Medical University, Xuzhou, China; ^4^ Department of Gastroenterology, The Affiliated Hospital of Xuzhou Medical University, Xuzhou, China; ^5^ Cancer Institute, Xuzhou Medical University, Xuzhou, China; ^6^ Jiangsu Bone Marrow Stem Cell Institute, Xuzhou Medical University, Xuzhou, China

**Keywords:** chimeric antigen receptor T cell, hematological toxicity, BCMA, CD19, multiple myeloma

## Abstract

Although chimeric antigen receptor T (CAR-T) cell therapy has been indicated to be effective in treating relapsed or refractory multiple myeloma (R/R MM), severe hematological toxicity (HT) remains an intractable issue. This study enrolled 54 patients with R/R MM following combined infusion of anti-CD19 and anti-BCMA CAR-T cells. The results showed that the rates of severe cytopenia were high, including severe neutropenia (28/54, 52%), severe anemia (15/54, 28%), and severe thrombocytopenia (18/54, 33%). Moreover, the incidence of prolonged HT (PHT) on Day 28 post-infusion was 52% (28/54), including 46% for severe neutropenia, 30% for severe anemia, and 31% for severe thrombocytopenia. Patients with PHT had a poorer median progression-free survival (PFS) and overall survival (OS) than patients without PHT (*P*=0.011; *P*=0.007). Furthermore, Cox regression analyses showed that PHT was an independent risk factor for PFS and OS. Univariate analyses showed that IFNγ (OR: 1.046; 95% CI: 1.002-1.093, *P*=0.042) and severe HT after lymphodepletion chemotherapy (OR: 0.082; 95% CI: 0.017-0.404; *P*=0.002) were independent risk factors for PHT. In conclusion, these results indicated that PHT was associated with poor outcomes following CAR-T-cell therapy in MM patients. Early detection and management of PHT would be beneficial for the prevention of life-threatening complications and improvement in the survival of patients after CAR-T-cell therapy.

**Clinical trial registration:**

This trial was registered on 1 May 2017 at http://www.chictr.org.cn as ChiCTR-OIC-17011272.

## 1. Introduction

Multiple myeloma (MM) is a hematological malignancy caused by the malignant proliferation of plasma cells in the bone marrow (1). It is now clinically treated with chemotherapy, autologous hematopoietic stem cell transplantation (Auto-HSCT), proteasome inhibitors, immunomodulatory drugs, and monoclonal antibodies, but it will eventually relapse. The treatment of relapsed and refractory (R/R) MM remains a challenge (2–5). In recent years, with the development of CAR-T-cell technology, targeted BCMA CAR-T-cell therapy as a new method for the treatment of R/R MM has achieved high response rates and has shown curative effects in clinical trials (6–8). In R/R MM patients, the complete response (CR) rate of BCMA-targeted CAR-T-cell therapy is as high as 70-95% (9–11). However, various adverse effects (AEs) remain unresolved, limiting the wide application of CAR-T-cell therapy (12).

To date, cytokine release syndrome (CRS) and neurotoxicity are the two most common toxicities after CAR-T-cell infusion (13). With supportive approaches, including tocilizumab, corticosteroids and anakinra, symptoms are resolved in most patients. Hematological toxicity (HT) is another common AE with an incidence of higher than 90% and is associated with dismal outcomes (14–16). For instance, Sarah and Wang et al. reported that prolonged HT (PHT) is associated with a shorter 1-year OS in patients with R/R diffuse large B-cell lymphoma and acute lymphoblastic leukemia (17, 18). However, there are relatively few large-sample studies on post-CAR-T-cell therapy PHT among patients with R/R MM, and studies on the correlation between PHT and prognosis are lacking.

Our previous studies confirmed that the combined infusion of humanized anti-CD19 and anti-BCMA CAR-T cells is feasible and that the majority of patients with R/R MM achieved high response rates (19, 20). Here, we systematically analyzed the correlation between PHT and prognosis among patients with R/R MM participating in a phase 1/2 clinical trial of CAR-T-cell therapy. Moreover, the risk factors affecting PHT were evaluated.

## 2. Methods

### 2.1 Patient selection

A total of 54 patients with R/R MM who underwent CAR-T-cell therapy between July 2017 and August 2020 were retrospectively reviewed. All patients were enrolled in phase 1/2 open-label single-center clinical trials of CAR-T-cell therapy targeting BCMA and CD19 (Chictr.org.cn ChiCTR-OIC-17011272). This study was conducted in full compliance with the ethical principles of the Declaration of Helsinki and was approved by the Ethics Committee of the Affiliated Hospital of Xuzhou University. Eligible patients had histologically or cytologically confirmed MM and met the International Myeloma Working Group (IMWG) diagnostic criteria for R/R MM (21). Patients were aged 18-69 years, with a life expectancy of 12 weeks or more and adequate organ function. Patients with uncontrollable infections, mental or psychological illnesses, severe allergies, or a history of severe allergies were excluded.

### 2.2 CAR-T-cell manufacturing and clinical protocol

Peripheral blood mononuclear cells of the enrolled patients were collected for CAR-T-cell production. The protocol for CAR-T-cell manufacturing in our center has been described previously (19, 20). Each individual was administered a single cycle of fludarabine (30 mg/m^2^ on Day -5 to -3)- and cyclophosphamide (750 mg/m^2^ on Day -5)-based conditioning treatment, followed by CAR-T-cell infusion. The established hospitalization observation time was 1 month, but it could change depending on the severity and the recovery of toxicity. The patients’ vital signs were monitored daily. Routine blood tests were conducted, and the levels of serum cytokines were determined by cytometric bead array (CBA) at least three times a week. Detailed information is provided in the **Supplementary material**.

### 2.3 Definitions of HT and hematologic recovery

The criteria for cytopenia and recovery were defined as per the Center for International Blood and Marrow Transplant Research (CIBMTR) (22) reporting guidelines. Neutropenia and severe neutropenia were defined as absolute neutrophil counts (ANC) lower than 1.5×10^9^/L and 0.5×10^9^/L, respectively. Anemia was defined as a hemoglobin concentration lower than 120 g/L in men and 110 g/L in women; levels lower than 60 g/L were considered severe anemia. Thrombocytopenia and severe thrombocytopenia were defined as platelet counts < 100×10^9^/L and < 20×10^9^/L, respectively. Neutrophil recovery was defined as an ANC > 0.5 ×10^9^/L for three consecutive days, irrespective of growth factor administration. Hemoglobin recovery was defined as a hemoglobin concentration > 60 g/L without the support of erythrocyte transfusion. Platelet recovery was defined as platelet counts > 20×10^9^/L for three consecutive days in the absence of platelet transfusion. PHT was defined as the presence of severe neutropenia, severe anemia, or severe thrombocytopenia on Day 28 post-infusion.

### 2.4 CRS and neurotoxicity

CRS effects were graded and managed according to the recommendations of Lee et al. (23). Grade 1-2 CRS was classified as “mild,” while grade 3-4 CRS was classified as “severe.” The assessment of neurotoxicity was based on the Common Terminology Criteria for Adverse Events 5.0 (CTCAE 5.0) (24). CRS and neurotoxicity, along with other factors, including clinical symptoms, vital signs, and levels of serum cytokines, were assessed by three experienced clinicians. Inconsistencies were further discussed.

### 2.5 Response to CAR T-cell therapy

The entire cohort was evaluated to assess the response to CAR-T-cell therapy. The response to treatment was evaluated on Day +90 using the International Myeloma Working Group criteria (21), including a stringent complete response (sCR), a complete response (CR), a very good partial response (VGPR), a partial response (PR), a minimal response (MR), stable disease (SD) and progressive disease (PD). The overall response rate (ORR) was defined as patients who achieved a PR or better. Overall survival (OS) was defined as the time from CAR-T-cell infusion censored on the date of the last follow-up or death from any cause. Progression-free survival (PFS) was calculated from the date of CAR-T-cell infusion to the date of disease progression (imaging or biopsy) or death from any cause.

### 2.6 Statistical analysis

Descriptive statistics were used to describe the patients’ baseline characteristics and the temporal profiles of severe cytopenia. Univariate and multivariate logistic regression models were applied to assess whether several variables were contributing factors to PHT. Categorical variables were analyzed using the chi-square test and Fisher’s exact test. Descriptive and survival analyses were performed using the Kaplan−Meier methodology. A log-rank test was utilized to compare OS and PFS between patient groups. Multivariate Cox regression models were used for the analysis of factors related to survival. A p value <0.05 was considered significant. Analysis was performed using GraphPad Prism software version 8.0.

## 3. Results

### 3.1 Patient characteristics

A total of 54 R/R MM patients treated with CAR-T-cell therapy between July 2017 and August 2020 at the Affiliated Hospital of Xuzhou Medical University were followed. Patients were divided into a PHT group (28 patients, 52%) and a non-PHT group (26 patients, 48%) based on the occurrence of PHT at 28 days after CAR-T-cell infusion. In the PHT group, the median age was 58 years (range, 30 to 67), the median time since diagnosis was 39 months (range, 8 to 167), and 9 patients (32%) with the high tumor burden at CAR T-cell infusion. Thirteen patients (46%) had International Staging System stage III disease, and 8 patients (29%) had extramedullary disease. High-risk cytogenetic abnormalities were detected in 8 patients (29%). Before the lymphodepletion regimen, the patients received a median of 4 (range, 2 to 17) previous therapy lines, and 10 patients (36%) had received autologous hematopoietic stem cell transplantation (ASCT). In the non-PHT group, the median age was 57 (range, 43 to 67), the median time of diagnosis was 40 months (range, 8 to 113) and 4 patients (15%) with the high tumor burden. Eleven patients (42%) had International Staging System stage III disease, and 7 patients (31%) had extramedullary lesions. Seven patients (27%) had high-risk cytogenetic abnormalities. The patients received a median of four chemotherapy cycles (range, 2 to 7), and 5 patients (19%) received ASCT before CAR T-cell infusion (**Table 1**).

**Table 1:** Demographic and clinical characteristics of the enrolled patients.

	All MM patients (N = 54)	Patients with PHT (N = 28)	Patients without PHT (N = 26)	*p*
Characteristic				
Age, y, median (range)	58 (30-67)	58 (30-67)	57(43-67)	0.812
Male sex - no. (%)	26 (48)	11 (39)	15 (58)	0.176
ISS stage - no. (%)				0.761
I or II	30 (56)	15 (54)	15 (58)	
III	24 (44)	13 (46)	11 (42)	
High tumor burden - no, (%) ¶	13 (24)	9 (32)	4 (15)	0.262
High-risk cytogenetic #	15 (28)	8 (29)	7 (27)	0.893
Extramedullary disease, no. (%)	15 (28)	8 (29)	7 (27)	0.893
Previous therapy lines, median (range)	4 (2-17)	4 (2-17)	4 (2-7)	0.083
Median time since diagnosis (mos., range)	40 (8-167)	39 (8-167)	40 (8-113)	0.901
Previous ASCT - no. (%)	15 (28)	10 (36)	5 (19)	0.177
Prelymphodepletion				
Median ANC, ×10^9^/L	2.11 (0.37-6.87)	1.82 (0.37-6.87)	2.42 (0.97-3.98)	
Median hemoglobin, g/L	99 (47-158)	91 (47-149)	108 (59-158)	
Median platelet count, ×10^9^/L	115 (13-329)	81 (13-230)	153 (79-329)	

Percentages may not total to 100 because of rounding

PHT, prolonged hematological toxicity; ISS, International Staging System; ASCT, autologous stem cell transplantation; ANC, absolute neutrophil count; HT, hematological toxicity; ORR, overall response rate; sCR, stringent complete response; CR, complete response; VGPR, very good partial response; PR, partial response; SD, stable disease. ¶ High tumor burden was defined as at least 50% clonal plasma cells or bone marrow plasma cells. # The cytogenetic risk profile was reported by investigators on the basis of fluorescence in situ hybridization. A high-risk cytogenetic profile was defined by the presence of the following abnormalities: del(17p), t (4;14), or t (14;16). Two-sided P values were calculated using the Mann–Whitney U test for continuous variables and Pearson’s chi-square or Fisher’s exact test for categorical variables.

### 3.2 Patient outcomes

#### 3.2.1 Response rates

**Figure 1:**
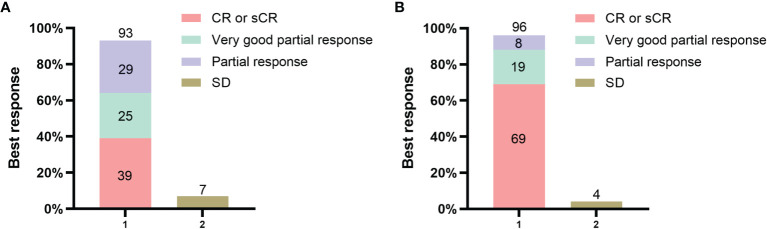
Response to the combination of anti-BCMA and anti-CD19 chimeric antigen receptor (CAR) T cells. Panel **(A)** shows the rates of overall response and no response in the PHT group. Panel **(B)** shows the rates of overall response and no response in the non-PHT group. All responses were confirmed and assessed on the basis of the International Myeloma Working Group Uniform (IMWG) Response Criteria for Multiple Myeloma.

In the PHT group, the ORR to CAR-T-cell therapy was 93% (26/28) 3 months after infusion. The best response to therapy was as follows: 6 patients achieved an sCR, 5 achieved a CR, 7 achieved a VGPR, 8 achieved a PR and 2 achieved SD (**Figure 1A**). In the non-PHT group, the ORR to CAR-T-cell therapy was 96% (25/26). The best response to therapy was as follows: 11 patients achieved an sCR, 7 achieved a CR, 5 achieved a VGPR, 2 achieved a PR and 1 achieved SD (**Figure 1B**). We found no significant difference in the ORR between the two groups.

#### 3.2.2 PFS and OS

**Figure 2:**
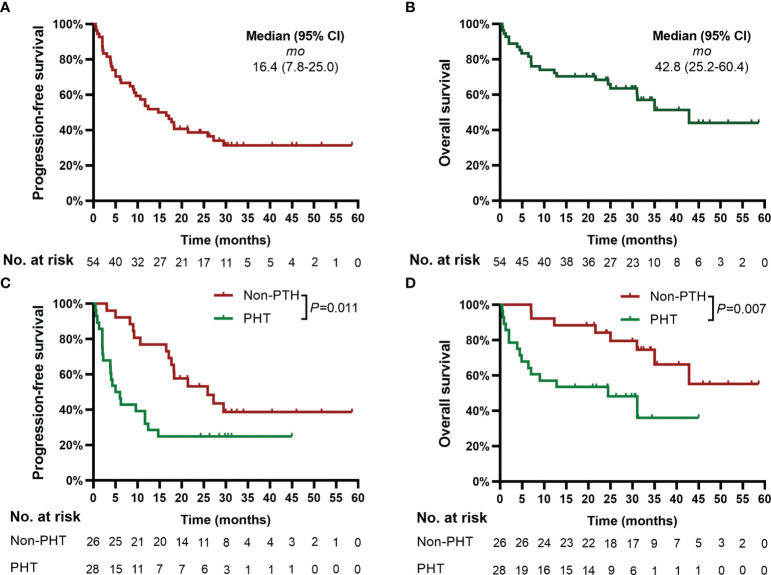
Progression-free survival (PFS) and overall survival (OS). Panels **(A, B)** show the Kaplan−Meier curves for PFS and OS in the 54 patients, respectively. Panels **(C, D)** show the Kaplan−Meier curves of PFS and OS, respectively, according to PHT. Two-sided P values were calculated based on the log-rank test. PHT, prolonged hematological toxicity.

The deadline of follow-up was April 30, 2022, with a median follow-up of 24.3 months (range, 0.5 to 58.6) after CAR-T-cell infusion. Based on Kaplan−Meier estimates, the median PFS and OS for 54 patients were 16.4 months (95% CI, 7.8 to 25.0) and 42.8 months (95% CI, 25.2 to 60.4), respectively (**Figures 2A, B**).

#### 3.2.3 Risk factors for PFS and OS

Subgroup analyses showed that patients’ baseline characteristics, including disease stage, extramedullary disease, time since diagnosis and previous therapy lines, were not associated with PFS or OS. Patients with a high baseline tumor burden (HR: 5.117; 95% CI: 2.340-11.451; *P*=0.001) and high-risk cytogenetic (HR: 2.278; 95% CI: 1.155-4.496; *P*=0.014) had a lower PFS than those with a low baseline tumor burden and no high-risk cytogenetic. Moreover, the severity of CRS (HR: 3.027; 95% CI: 1.115-8.214; *P*=0.022) was significantly associated with OS (**Figure S1**).

#### 3.2.4 Prognosis

In the PHT group, 21 patients (75%) relapsed, and 15 (54%) died during follow-up. Among these patients, 10 patients (36%) died from disease progression or associated complications. Two patients (7%) died from intracranial hemorrhage, 2 (7%) died from severe infection, and 1 (4%) died from severe CRS within 2 months. In the non-PHT group, 15 patients (58%) relapsed, and 8 (31%) died, all of whom died from disease progression or associated complications (**Table 2**).

**Table 2:** PHT with other adverse events and interventions after CAR-T-cell infusion.

Characteristic	PHT (n = 28)	Non-PHT (n = 26)	P value
Adverse events			
CRS, no. (%)			0.480
Grade 1-2	23 (82)	24 (92)	
Grade 3-5	5 (18)	2 (8)	
Median time to CRS onset, median (range)	6 (0-13)	7 (0-13)	0.875
Median duration, median (range)	5 (1-20)	4 (1-8)	0.463
Neurotoxicity, no. (%)	2 (7)	0	0.491
Infection, n (%)	18 (64)	8 (31)	0.014
Unspecified pathogen	6 (21)	1 (4)	
Viral	4 (14)	3 (12)	
Bacterial	9 (32)	4 (15)	
Fungal	2 (7)	1 (4)	
Hemorrhage, n (%)	2 (7)	0	0.491
Interventions			
G-CSF, n (%)	27 (96)	18 (69)	0.021
PRBC transfusions, n (%)	17 (61)	2 (8)	< 0.001
Platelet transfusions, n (%)	16 (57)	2 (8)	< 0.001

PHT, prolonged hematological toxicity; CRS, cytokine release syndrome; G-CSF, granulocyte colony-stimulating factor; PRBC, packed red blood cell. Data are described as n (%). P values were tested by the chi-square test and Fisher’s exact test for categorical variables.

### 3.3 Adverse events and interventions

#### 3.3.1 CRS and neurotoxicity

In the PHT group, 23 patients (82%) developed mild CRS, and 5 patients (18%) developed severe CRS. The median time to CRS onset was 6 days (range 0-13), with a median duration of 5 days (range 1-12). Twelve patients (43%) received tocilizumab, and 17 patients (61%) received glucocorticoids. Moreover, 2 patients (7%) developed grade 3 and grade 4 neurotoxicity. In the non-PHT group, 24 patients (92%) sustained mild CRS, and 2 patients (8%) sustained severe CRS. The median time to CRS onset was 7 days (range 0-13), with a median duration of 4 days (range 1-8). Four patients (15%) received tocilizumab, and 6 patients (23%) received glucocorticoids. None of the patients developed neurotoxicity (**Table 2**).

#### 3.3.2 Incidence and temporal characteristics of HT

**Figure 3:**
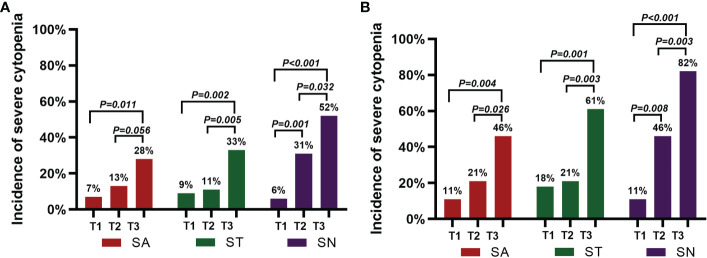
Incidence of severe cytopenia before and after CAR-T-cell infusion. **(A)** shows the percentage of severe cytopenia changes after lymphodepletion chemotherapy and CAR-T-cell infusion in the 54 patients. **(B)** shows the percentage of severe cytopenia changes after lymphodepletion chemotherapy and CAR-T-cell infusion in the 28 patients with PHT. A two-sided P value was determined *via* the Pearson chi-square test.

Before lymphodepleting chemotherapy, in the PHT group, the median ANC was 1.82 (range, 0.37-6.87) ×10^9^/L, the median hemoglobin serum concentration was 91 (range, 47-149) g/L, and the median platelet count was 81 (range, 13-230) ×10^9^/L. After lymphodepletion chemotherapy, the incidence of severe neutropenia significantly increased from 11% to 46% (P<0.05), while no significant changes were detected with respect to severe anemia (11% *vs.* 21%) or severe thrombocytopenia (18% *vs.* 21%). After CAR-T-cell infusion, the total incidence of HT increased remarkably (82% for severe neutropenia, 46% for severe anemia, and 61% for severe thrombocytopenia, P<0.05) (**Figure 3**). Moreover, 27 (96%) patients received granulocyte colony-stimulating factor (G-CSF), 17 (61%) received packed red blood cell (PRBC) transfusions, and 16 (57%) received platelet transfusions (**Table 2**). In the non-PHT group, the median ANC was 2.42 (range, 0.97-3.98) ×10^9^/L, the median hemoglobin serum concentration was 108 (range, 59-158) g/L, and the median platelet count was 153 (range, 79-329) ×10^9^/L. After lymphodepletion chemotherapy, no significant changes were detected with respect to severe neutropenia (0% *vs.* 15%), severe anemia (4% *vs.* 4%) or severe thrombocytopenia (0% *vs.* 0%). After CAR-T-cell infusion, the total incidence of HT did not increase remarkably (19% for SN, 4% for severe anemia, and 0% for severe thrombocytopenia). Among these patients, 18 (69%) received G-CSF, 2 (8%) received PRBC transfusions and 2 (8%) received platelet transfusions. Of note, compared with PHT patients, patients without PHT showed a lower requirement for blood transfusion or G-CSF support (P<0.005) (**Table 2**). In the PHT group and non-PHT group, the median time from infusion to recovery from neutropenia, anemia and thrombocytopenia was 42 *vs.* 15 days (range 10-159 *vs.* 6-71), 53 *vs.* 34 days (range 9-213 *vs.* 10-90) and 53 *vs.* 21 days (range 7-210 *vs.* 6-45), respectively.

#### 3.3.3 Infection

In the PHT group, 18 of the 28 patients (64%) experienced infections, mainly including 10 (36%) with lung infections, 2 (7%) with upper respiratory tract infections, 2 (7%) with bacteremia, 1 (4%) with a urinary tract infection and 3 (11%) with skin soft-tissue infections after CAR-T-cell infusion. Moreover, 9 patients (32%) had bacterial infections, 2 (7%) had fungal infections, 2 (7%) had herpes zoster virus infections, 1 (4%) had a hepatitis B virus infection, 1 (4%) had hepatitis B virus activation, and 6 (21%) had unspecified pathogen infections. In the non-PHT group, 8 patients (31%) were infected with 4 (15%) lung infections, 1 (4%) bacteremia infection and 2 (8%) urinary tract infections. Among these patients, 4 patients (15%) had bacterial infections, 1 (4%) had a fungal infection, 1 (4%) had a herpes zoster virus infection, 1 (4%) had a hepatitis B virus infection, 1 (4%) had a cytomegalovirus infection and 1 (4%) had an unspecified pathogen infection (**Table 2**).

### 3.4 PHT with PFS and OS

Patients with PHT had significantly poorer PFS (median of 5.0 months [95% CI, 2.1 to 7.9] *vs.* 45.9 months [95% CI, 13.4 to 38.4], *P*=0.011) and OS (median of 24.5 months [95% CI, 4.1 to 44.9] *vs.* not reached, *P*=0.007) than patients without PHT (**Figures 2C, D**).

Further analysis demonstrated that the patients with severe neutropenia, severe anemia and severe thrombocytopenia had an inferior PFS (severe neutropenia: median of 5.0 months [95% CI, 1.7 to 8.3] *vs.* 25.9 months [95% CI, 13.0 to 38.8], *P*=0.010; severe anemia: median of 2.2 months [95% CI, 0.0 to 5.5] *vs.* 18.2 months [95% CI, 11.9 to 24.5], *P*=0.018; severe thrombocytopenia: median of 3.8 months [95% CI, 1.4 to 6.2] *vs.* 25.9 months [95% CI, 14.3 to 37.5], *P<*0.001) and OS (severe neutropenia: median of 24.5 months [95% CI, 16.2 to 32.0] *vs.* not reached, *P*=0.018; severe anemia: median of 4.9 months [95% CI, 0.0 to 9.8] *vs.* 42.8 months [95% CI, 27.0 to 58.6], *P*=0.009; severe thrombocytopenia: median of 6.4 months [95% CI, 3.0 to 9.8] *vs.* not reached, *P<*0.001) than patients without severe neutropenia, severe anemia and severe thrombocytopenia at 28 days after CAR-T-cell infusion (**Figure S2**).

Furthermore, Cox regression analyses showed that PHT (HR: 2.762; 95% CI: 1.355-5.631; *P*=0.005), baseline tumor burden (HR: 3.635; 95% CI: 1.498-8.821; *P*=0.004) and having high-risk cytogenetic (HR: 2.945; 95% CI: 1.387-6.255; *P*=0.005) were independent risk factors for PFS. Moreover, PHT (HR: 3.347; 95% CI: 1.318-8.503; *P*=0.011) and the severity of CRS (HR: 3.084; 95% CI: 1.004-9.474; *P*=0.049) were also independent risk factors for OS (**Tables S1, 2**).

### 3.5 Factors associated with the incidence of PHT

Next, we analyzed the patients’ characteristics, prior therapies, serum cytokine levels and CAR-T-cell therapy-associated factors to identify the risk factors correlated with PHT.

Univariate analyses revealed that the severe HT after lymphodepletion chemotherapy (OR: 8.500; 95% CI: 2.299-31.431; *P*=0.001), grade of CRS (OR: 2.697; 95% CI: 1.152-6.312; *P*=0.022) and the levels of several serum biomarkers (including peak levels of IL-6 (OR: 1.008; 95% CI: 1.001-1.015; *P*=0.025), IL-8 (OR: 1.011; 95% CI: 1.002-1.020; *P*=0.019), IFNγ (OR: 1.036; 95% CI: 1.002-1.072; *P*=0.040), and MIP1α (OR: 1.066; 95% CI: 1.000-1.137; *P*=0.049) were significantly associated with the incidence of PHT. Multivariate analysis revealed that IFNγ (OR: 1.046; 95% CI: 1.002-1.093, *P*=0.042) and severe HT after lymphodepletion chemotherapy (OR: 0.054; 95% CI: 0.008-0.357; *P*=0.002) were independent risk factors for PHT (**Table 3**).

**Table 3:** Factors related to PHT.

Factor	Univariate Analysis	Multivariate Analysis		
	P value *	Hazard Ratio	95% CI	P value
ISS Stage III	0.761			
High-risk **cytogenetic** #	0.675			
Previous ASCT	0.182			
Extramedullary disease	0.893			
High tumor burden¶	0.158	1.739	0.259-11.681	0.569#
Time since diagnosis	0.635			
≥5 yrs. versus <5 yrs.				
Previous therapy lines	0.219			
>3 versus ≤ 3				
Severe HT after lymphodepletion chemotherapy	0.001	0.054	0.008-0.457	0.002#
Severity of CRS	0.279			
Grade of CRS	0.022	2.099	0.608-7.253	0.241#
Cytokines, (pg/mL)				
IL-6	0.025	1.006	0.997-1.015	0.198#
IL-8	0.019	1.007	0.990-1.024	0.450#
IFN γ	0.040	1.046	1.002-1.093	0.042#
MIP1α	0.049	1.026	0.964-1.092	0.425#

PHT, prolonged hematological toxicity; CI, confidence interval; ISS, International Staging System, ASCT, autologous stem cell transplantation; HT, hematological toxicity; CRS, cytokine release syndrome. # The cytogenetic risk profile was reported by investigators on the basis of fluorescence in situ hybridization. A high-risk cytogenetic profile was defined by the presence of the following abnormalities: del(17p), t (4;14), or t (14;16). ¶ High tumor burden was defined as at least 50% clonal plasma cells or bone marrow plasma cells. * Two-sided P values were calculated on the basis of logistic regression. # A logistic regression model was used for multivariate analysis. The variables in which the P value was ≤ 0.1 by univariate analysis or the variables that may have affected the results were included.

## 4. Discussion

In our previous study, 21 patients with R/R MM received an infusion of anti-BCMA and humanized anti-CD19 CAR-T cells. At a median follow-up of 179 days, 20 (95%) of the 21 patients had an overall response. In this retrospective study, we further expanded the sample size and demonstrated that 51 (95%) of 54 patients achieved a PR or better. Moreover, the occurrence of severe CRS and neurotoxicity was relatively low. These results indicated that the combined infusion of anti-BCMA and anti-CD19 CAR-T cells is feasible for patients with R/R MM.

During the management of patients with R/R MM who are receiving CAR-T-cell therapy, HT, in addition to CRS and neurotoxicity, is a major issue for clinicians and has a higher incidence. In the phase 2 study, for the 128 patients with R/R MM following idecabtagene vicleucel infusion (25), the incidences of HT were 89% for severe neutropenia, 60% for severe anemia and 52% for severe thrombocytopenia. Among all patients, 41% (52 patients) had persistent severe neutropenia, and 48% (62 patients) had persistent severe thrombocytopenia 1 month after infusion. In the phase 1 study, for the 33 patients with R/R MM following bb2121 infusion (6), the incidences of HT were 85% for severe neutropenia, 45% for severe anemia and 45% for severe thrombocytopenia, and PHT, with incidences of 3% for severe neutropenia and 35% for severe thrombocytopenia, was not resolved by Day +28 after cell therapy. In the phase 1 dose-climbing and expansion study following a bispecific CAR-T-cell therapy targeting BCMA and CD38 (BM38) in 23 patients with R/R MM (26), HT was the most common adverse event; severe neutropenia occurred in 83% of the patients, severe anemia occurred in 13% and severe thrombocytopenia occurred in 48%, with 40% of the patients with severe neutropenia and 55% with severe thrombocytopenia not having recovered within 1 month. In our studies, the incidences of HT were 52% for severe neutropenia, 28% for severe anemia and 33% for severe thrombocytopenia. Moreover, 46% of the patients with severe neutropenia, 30% with severe anemia and 31% with severe thrombocytopenia were not recovered by Day +28, which is consistent with the above reported study.

Previous studies have shown that patients with PHT have a poor prognosis after CAR-T-cell infusion. Sarah et al. (17) reported that the 1-year PFS and 1-year OS in patients with PHT were 24% and 36%, respectively, in 31 patients with R/R diffuse large B-cell lymphoma (DLBCL) who received tisagenlecleucel or axicabtagene ciloleucel. Moreover, patients without PHT had a longer 1-year OS of 81%. In a phase 1/2 study of 86 patients with R/R ALL who underwent CD19 CAR-T-cell therapy, the results demonstrated that persistent severe cytopenia was highly associated with a poor 1-year OS. However, in R/R MM patients, the correlation between PHT and prognosis is rarely reported. Notably, our results showed that patients with PHT had significantly poorer median PFS and OS than those without PHT. Moreover, patients with severe neutropenia, severe anemia, or severe thrombocytopenia at 28 days also had a shorter PFS and OS. Multivariate analysis revealed that PHT was an independent risk factor for poor PFS and OS. Therefore, additional measures are required to reduce PHT incidence after CAR-T-cell infusion to improve survival.

Prolonged cytopenias after CAR-T therapy have increasingly been reported at varying rates, and the pathogenesis of this complication is not yet well-understood but is likely contributed to by multiple factors. Sarah et al. (17). showed that the development of CRS, the administration of tocilizumab or steroids, and the levels of ferritin and CRP were positively associated with the occurrence of PHT in R/R DLBCL. Wang et al. (18). found that the baseline bone marrow tumor burden, CRS severity, and serum biomarker levels (including max lg CRP, IL-10, IFNγ, ferritin, and D-dimer levels) were associated with the incidence of PHT in R/R ALL. We also further analyzed the risk factors for PHT and found that severe HT after lymphodepletion chemotherapy, levels of serum cytokines, grade of CRS and infection were involved in the occurrence of PHT. However, the severity of CRS, time to CRS onset and duration of CRS were not related to PHT, which is inconsistent with previous studies. This may be related to the lower incidence of severe CRS in our trial. Therefore, some possible measures to reduce severe HT after lymphodepletion chemotherapy should be considered, such as the optimization of the lymphodepletion chemotherapy and early application of promoting blood cell growth, including the usage of G-CSF, blood transfusions, antibiotics and so on. In this trial, patients with severe hematological toxicity received G-CSF, blood transfusions, and other treatments that improved the degree of cytopenia. However, whether the application of these supportive treatments can affect the long-term prognosis of patients will be an issue to be investigated after we expand the sample size.

Our study is limited because it was a retrospective single-center study based on a relatively large sample size and longer follow-up time. We demonstrated that the combined infusion of humanized anti-CD19 and anti-BCMA CAR-T-cells was feasible, safe and significantly effective in treating patients with R/R MM. Moreover, HT remains one of the most common AEs after CAR-T-cell infusion, and the occurrence of PHT is associated with a poor prognosis in patients with R/R MM. Accordingly, enhancing bridging therapy to reduce the baseline tumor burden, monitoring serum biomarker levels, optimizing lymphodepletion chemotherapy and providing appropriate supportive treatment may be essential to reduce the incidence of PHT and improve the outcome of CAR-T-cell therapy in R/R MM patients. The preliminary results need to be confirmed in future prospective and multicenter clinical trials, and the mechanism of PHT after CAR-T-cell infusion requires further exploration.

## Data availability statement

The raw data supporting the conclusions of this article will be made available by the authors, without undue reservation.

## Ethics statement

The studies involving human participants were reviewed and approved by the Ethics Committee of the Affiliated Hospital of Xuzhou University. The patients/participants provided their written informed consent to participate in this study.

## Author contributions

HL, LZh, and ZS wrote the manuscript; LZ, YY, JW, TH, and SJ acquired the data; LZh, SW, ZS, HC, and MS interpreted the data and performed the statistical analyses. LL, LZe, QW, JQ, CC, KX, JZ, and JC helped revise the manuscript. All authors contributed to the article and approved the submitted version.

## Funding

This work was financially supported by grants from the Key Research & Development Plan of Jiangsu Province (BE2018634, BE2022711), Xuzhou Medical leading talents Training program (XWRCHT20210028), and Key Research & Development Plan of Xuzhou (KC18102, KC21185).

## Acknowledgments

We thank the study participants and their families and the staff of the Department of Hematology at the Affiliated Hospital of Xuzhou Medical University.

## Conflict of interest

The authors declare that the research was conducted in the absence of any commercial or financial relationships that could be construed as a potential conflict of interest.

## Publisher’s note

All claims expressed in this article are solely those of the authors and do not necessarily represent those of their affiliated organizations, or those of the publisher, the editors and the reviewers. Any product that may be evaluated in this article, or claim that may be made by its manufacturer, is not guaranteed or endorsed by the publisher.

## References

[1] Hosen N . Chimeric antigen receptor T-cell therapy for multiple myeloma. Int J Hematol (2020) 111(4):530–4. doi: 10.1007/s12185-020-02827-8 31981097

[2] Palumbo A Chanan-Khan A Weisel K Nooka AK Masszi T Beksac M . Daratumumab, bortezomib, and dexamethasone for multiple myeloma. N Engl J Med (2016) 375(8):754–66. doi: 10.1056/NEJMoa1606038 27557302

[3] McCarthy PL Owzar K Hofmeister CC Hurd DD Hassoun H Richardson PG . Lenalidomide after stem-cell transplantation for multiple myeloma. N Engl J Med (2012) 366(19):1770–81. doi: 10.1056/NEJMoa1114083 PMC3744390 22571201

[4] Krishnan A Pasquini MC Logan B Stadtmauer EA Vesole DH Alyea E 3rd . Autologous haemopoietic stem-cell transplantation followed by allogeneic or autologous haemopoietic stem-cell transplantation in patients with multiple myeloma (BMT CTN 0102): a phase 3 biological assignment trial. Lancet Oncol (2011) 12(13):1195–203. doi: 10.1016/S1470-2045(11)70243-1 PMC3611089 21962393

[5] Kumar SK Dimopoulos MA Kastritis E Terpos E Nahi H Goldschmidt H . Natural history of relapsed myeloma, refractory to immunomodulatory drugs and proteasome inhibitors: a multicenter IMWG study. Leukemia (2017) 31(11):2443–8. doi: 10.1038/leu.2017.138 28620163

[6] Raje N Berdeja J Lin Y Siegel D Jagannath S Madduri D . Anti-BCMA CAR T-cell therapy bb2121 in relapsed or refractory multiple myeloma. N Engl J Med (2019) 380(18):1726–37. doi: 10.1056/NEJMoa1817226 PMC8202968 31042825

[7] Davis JA Shockley A Hashmi H . The emergence of b-cell maturation antigen (BCMA) targeting immunotherapy in multiple myeloma. J Oncol Pharm Pract (2022) 28(4):960–8. doi: 10.1177/10781552211073517 35006032

[8] Anderson LD Jr . Idecabtagene vicleucel (ide-cel) CAR T-cell therapy for relapsed and refractory multiple myeloma. Future Oncol (2022) 18(3):277–89. doi: 10.2217/fon-2021-1090 34854741

[9] Xu J Chen LJ Yang SS Sun Y Wu W Liu YF . Exploratory trial of a biepitopic CAR T-targeting b cell maturation antigen in relapsed/refractory multiple myeloma. Proc Natl Acad Sci U S A (2019) 116(19):9543–51. doi: 10.1073/pnas.1819745116 PMC6510991 30988175

[10] Cohen AD Garfall AL Stadtmauer ED Melenhorst JJ Lacey SF Lancaster E . B cell maturation antigen-specific CAR T cells are clinically active in multiple myeloma. J Clin Invest (2019) 129(6):2210–21. doi: 10.1172/JCI126397 PMC6546468 30896447

[11] Brudno JN Maric I Hartman SD Rose JJ Wang M Lam N . T Cells genetically modified to express an anti-B-Cell maturation antigen chimeric antigen receptor cause remissions of poor-prognosis relapsed multiple myeloma. J Clin Oncol (2018) 36(22):2267–80. doi: 10.1200/JCO.2018.77.8084 PMC6067798 29812997

[12] Rafiq S Hackett CS Brentjens RJ . Engineering strategies to overcome the current roadblocks in CAR T cell therapy. Nat Rev Clin Oncol (2020) 17(3):147–67. doi: 10.1038/s41571-019-0297-y PMC7223338 31848460

[13] Brudno JN Kochenderfer JN . Recent advances in CAR T-cell toxicity: Mechanisms, manifestations and management. Blood Rev (2019) 34:45–55. doi: 10.1016/j.blre.2018.11.002 30528964 PMC6628697

[14] Nahas GR Komanduri KV Pereira D Goodman M Jimenez AM Beitinjaneh A . Et al: Incidence and risk factors associated with a syndrome of persistent cytopenias after CAR-T cell therapy (PCTT). Leuk Lymphoma (2020) 61(4):940–3. doi: 10.1080/10428194.2019.1697814 31793821

[15] Jain T Knezevic A Pennisi M Chen Y Ruiz JD Purdon TJ . Et al: Hematopoietic recovery in patients receiving chimeric antigen receptor T-cell therapy for hematologic malignancies. Blood Adv (2020) 4(15):3776–87. doi: 10.1182/bloodadvances.2020002509 PMC7422135 32780846

[16] Fried S Avigdor A Bet B Meir A MJ B Schachter J . Early and late hematologic toxicity following CD19 CAR-T cells. Bone Marrow Transpl (2019) 54(10):1643–50. doi: 10.1038/s41409-019-0487-3 30809033

[17] Nagle SJ Murphree C Raess PW Schachter L Chen A Hayes-Lattin B . Prolonged hematologic toxicity following treatment with chimeric antigen receptor T cells in patients with hematologic malignancies. Am J Hematol (2021) 96(4):445–61. doi: 10.1002/ajh.26113 33529419

[18] Wang L Hong R Zhou L Ni F Zhang M Zhao H . New-onset severe cytopenia after CAR-T cell therapy: Analysis of 76 patients with relapsed or refractory acute lymphoblastic leukemia. Front Oncol. 11:702644. doi: 10.3389/fonc.2021.702644 PMC8278328 34277448

[19] Yan Z Cao J Cheng H Qiao J Zhang H Wang Y . A combination of humanised anti-CD19 and anti-BCMA CAR T cells in patients with relapsed or refractory multiple myeloma: a single-arm, phase 2 trial. Lancet Haematol (2019) 6(10):e521–9. doi: 10.1016/S2352-3026(19)30115-2 31378662

[20] Wang Y Cao J Gu W Shi M Lan J Yan Z . Long-term follow-up of combination of b-cell maturation antigen and CD19 chimeric antigen receptor T cells in multiple myeloma. J Clin Oncol (2022) 40(20):2246–56. doi: 10.1200/JCO.21.01676 35333600

[21] Palumbo A Rajkumar SV San Miguel JF Larocca A Niesvizky R Morgan G . International myeloma working group consensus statement for the management, treatment, and supportive care of patients with myeloma not eligible for standard autologous stem-cell transplantation. J Clin Oncol (2014) 32(6):587–600. doi: 10.1200/JCO.2013.48.7934 24419113 PMC3918540

[22] Center for International Blood & Marrow Transplant Research . Cellular therapy manuals - peripheral blood count recovery. Available at: https://www.cibmtr.org/ReferenceCenter/SlidesReports/Pages/index.aspx.

[23] Lee DW Gardner R Porter DL Louis CU Ahmed N Jensen M . Current concepts in the diagnosis and management of cytokine release syndrome. Blood (2014) 124(2):188–95. doi: 10.1182/blood-2014-05-552729 PMC4093680 24876563

[24] National Cancer Institute . Common terminology criteriafor adverse events (CTCAE). version 5.0 . Available at: https://ctep.cancer.gov/protocolDevelopment/electronic_applications/docs/CTCAE_v5_Quick_Reference_8.5x11.pdf.

[25] Mei H Li C Jiang H Zhao X Huang Z Jin D . A bispecifc CAR-T cell therapy targeting BCMA and CD38 in relapsed or refractory multiple myeloma. J Hematol Oncol (2021) 14(1):161. doi: 10.1186/s13045-021-01170-7 34627333 PMC8501733

[26] Munshi NC Anderson LD Jr Shah N Madduri D Berdeja J Lonial S . Idecabtagene vicleucel in relapsed and refractory multiple myeloma. N Engl J Med (2021) 384(8):705–16. doi: 10.1056/NEJMoa2024850 33626253

