# Supplementary material for: Prolonged hematological toxicity in patients receiving BCMA/CD19 CAR-T-cell therapy for relapsed or refractory multiple myeloma
Source: Front Immunol. 2022 Oct 18;13:1019548. doi: 10.3389/fimmu.2022.1019548 (PMC9623176; doi:10.3389/fimmu.2022.1019548)
Supplement: Supplementary file 1 [file DataSheet_1.doc]

**Supplementary material**

**Prolonged hematological toxicity is associated with poor outcomes following CAR-T cell therapy in patients with relapsed or refractory multiple myeloma**

**Table of Contents**

[Supplemental Figures................................................................................................](#__RefHeading___Toc90582020)..................................1

[Supplemental Figure 1. Subgroup Analysis of PFS and OS.](#__RefHeading___Toc90582026)...............................................................1

[Supplemental Figure 2. Subgroup Analysis of PHT with PFS and OS.](#__RefHeading___Toc90582026)..............................................3

[Supplemental Tables....................................................................................................................................4](#__RefHeading___Toc90582030)

[Supplemental Table 1. Factors related to PFS.](#__RefHeading___Toc90582033)....................................................................................4

[Supplemental Table 2. Factors related to OS...................................................................................](#__RefHeading___Toc90582034)....5

Protocol………………………………......…………………………….........................……….................6

Abstract………………………………………………………...........................………………….......7

Background………………………………………………………………...........................………....10

Previous research………………………………………………………………..................………....11

Objective………………………………………………………………...........................………........17

Study Design, Principles and Test Procedures……………………...………… ……………..............17

Clinical efficacy evaluation…………………………………………………...........................……....24

Project risk………………………………………………………………….........................................25

Risk disposal SOP……………………………………………………………...............…………......29

Regulation………………………………………………………………………………......................33

Faculty…………………………………………………………………………...................................35

References…………………………………………..…………………………...................................36

Appendix…………………………………………………………………………….................................39

Appendix 1 Revised International Myeloma Working Group diagnostic criteria for multiple myeloma

and smouldering multiple myeloma…................................................................................39

Appendix 2 Karnofsky performance status scale definitions rating (%) criteria ……………….…....40

Appendix 3 CAR-T proliferation………………………………………………………………….........41

Appendix 4 Response criteria for Multiple Myeloma ………………………………….………...........42

Appendix 5 Response criteria for Multiple Myeloma (Relapsed)……………….…………………......44

Appendix 6 MRD………………………………………….....................................................................45

Appendix 7 CRS Toxicity Grading……………………..........................................................................46

Appendix 8 Staging systems for Multiple Myeloma ……......................................................................47

Appendix 9 CRS clinical observation …….............................................................................................48

Appendix 10 SOP of Serious Adverse Reactions during CART Treatment……….................................49

**Supplemental Figures**


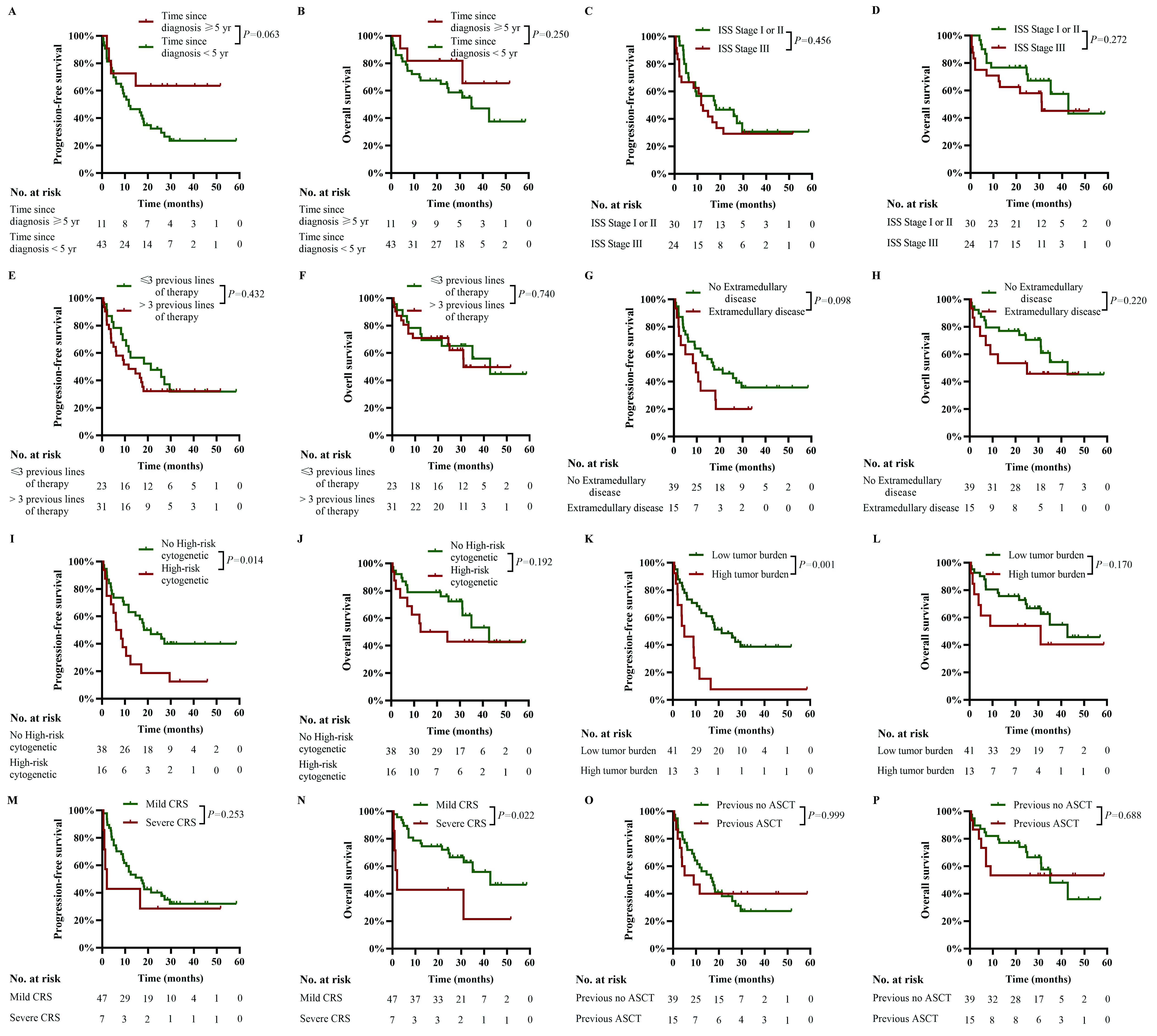


**Figure S1. Subgroup Analysis of PHT with PFS and OS.** Panels A and B show Kaplan-Meier curves of PFS and OS, respectively, according to the time since diagnosis. Panels C and D show Kaplan-Meier curves of PFS and OS, respectively, according to ISS stage. Panels E and F show Kaplan-Meier curves of PFS and OS, respectively, according to previous therapy lines. Panels G and H show Kaplan-Meier curves of PFS and OS, respectively, according to extramedullary disease. Panels I and J show Kaplan-Meier curves of PFS and OS, respectively, according to high-risk cytogenetics abnormality. High-risk cytogenetic profile was defined by the presence of the following abnormalities: del(17p), t (4;14), or t (14;16). Panels K and L show Kaplan-Meier curves of PFS and OS, respectively, according to tumor burden. High tumor burden was defined as at least 50% clonal plasma cells or bone marrow plasma cells. Panels M and N show Kaplan-Meier curves of PFS and OS, respectively, according to severity of CRS. Panels O and P show Kaplan-Meier curves of PFS and OS, respectively, according to previous ASCT. Tick marks indicate the time of data censoring at the last follow-up. ISS, International Staging System.


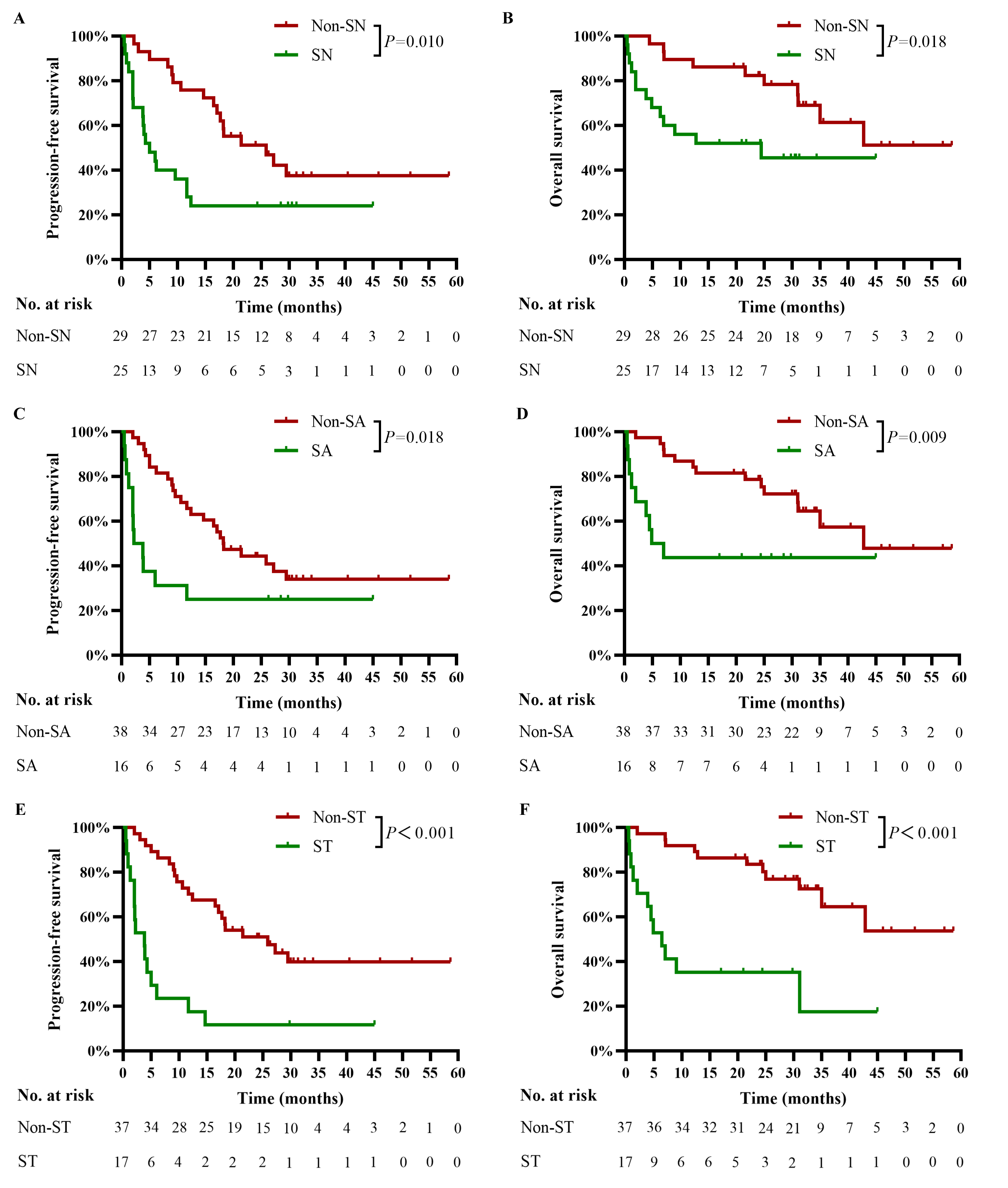


**Figure S2.** **Subgroup Analysis of PHT with PFS and OS.** Panel A and B shows Kaplan-Meier curves of PFS and OS, according to SN, respectively. Panel C and D shows Kaplan-Meier curves of PFS and OS, according to SA, respectively. Panel E and F shows Kaplan-Meier curves of PFS and OS, according to ST, respectively. Two-sided P-values calculated based on Log-rank test. SN, severe neutropenia; SA, severe anemia; ST, severe thrombocytopenia.

**Supplemental Tables**

**Table S1.** **Factors related to** **PFS.**

| Factors | Univariate Analysis | Multivariate Analysis | | |
| --- | --- | --- | --- | --- |
|  | P value * | Hazard Ratio | 95% CI | P value |
| ISS Stage Ⅲ | 0.456 |  |  |  |
| High-risk genetics £ | 0.014 | 2.945 | 1.387-6.255 | 0.005# |
| Previous ASCT | 0.999 | 0.597 | 0.250-1.425 | 0.245# |
| Extramedullary disease | 0.098 | 1.217 | 0.563-2.629 | 0.618# |
| High tumor burden | 0.001 | 3.635 | 1.498-8.821 | 0.004# |
| Time since diagnosis | 0.063 | 0.547 | 0.185-1.617 | 0.275# |
| ≥5 yr versus <5 yr |  |  |  |  |
| Previous therapy lines | 0.432 |  |  |  |
| >3 versus ≤ 3 |  |  |  |  |
| Infection | 0.107 |  |  |  |
| Severity of CRS | 0.253 |  |  |  |
| PHT | 0.011 | 2.762 | 1.355-5.631 | 0.005# |

Abbreviations: CI, confidence interval; ISS, International Staging System, ASCT, autologous stem cell transplantation; CRS, cytokine release syndrome; PHT, prolonged hematological toxicity.

£ The cytogenetic risk profile was reported by investigators on the basis of Fluorescence in-situ hybridization. High-risk cytogenetic profile was defined by the presence of the following abnormalities: del(17p), t (4;14), or t (14;16).

* Two-sided P-values were calculated on the basis of log-rank test.

# Cox regression model was used for multivariate analysis. The variables in which P value was ≤ 0.1 by univariate analysis or the variables that may affect the results were included.

**Table S2. Factors related to OS.**

| Factors | Univariate Analysis | Multivariate Analysis | | |
| --- | --- | --- | --- | --- |
|  | P value * | Hazard Ratio | 95% CI | P value |
| ISS Stage Ⅲ | 0.272 |  |  |  |
| High-risk genetics £ | 0.192 |  |  |  |
| Previous ASCT | 0.688 |  |  |  |
| Extramedullary disease | 0.220 |  |  |  |
| High tumor burden | 0.170 |  |  |  |
| Time since diagnosis | 0.250 |  |  |  |
| ≥5 yr versus <5 yr |  |  |  |  |
| Previous therapy lines | 0.740 |  |  |  |
| >3 versus ≤ 3 |  |  |  |  |
| Infection | 0.099 | 1.186 | 0.466-3.016 | 0.720# |
| Severity of CRS | 0.022 | 3.084 | 1.004-9.474 | 0.049# |
| PHT | 0.007 | 3.347 | 1.318-8.503 | 0.011# |

Abbreviations: CI, confidence interval; ISS, International Staging System, ASCT, autologous stem cell transplantation; CRS, cytokine release syndrome; PHT, prolonged hematological toxicity.

£ The cytogenetic risk profile was reported by investigators on the basis of Fluorescence in-situ hybridization. High-risk cytogenetic profile was defined by the presence of the following abnormalities: del(17p), t (4;14), or t (14;16).

* Two-sided P-values were calculated on the basis of log-rank test.

# Cox regression model was used for multivariate analysis. The variables in which P value was ≤ 0.1 by univariate analysis or the variables that may affect the results were included.

**Protocol**

**PI/Department:** Xu Kai-lin, M.D. & Ph.D./Department of hematology

**Funding Sponsor:**

**Study sponsor and monitor:** the Affiliated Hospital of Xuzhou Medical University

**Study duration:** 2017.05-2021.05

**Version:** V1.0

**Protocol updated:** 2017.03.1

**Abstract**

| **Title** | An open single center single arm clinical study of through infusion of anti-BCMA CAR-T and anti-CD19 CAR-T therapy for relapsed and refractory multiply myeloma |
| --- | --- |
| **Phase** | Phase I/II |
| **Study Design** | This is a single-center, open-label and single-arm study to assess the safety and efficacy of sequential infusion of anti-BCMA CAR-T and anti-CD19 CAR-T for relapsed and refractory multiple myeloma |
| **Number of subjects** | 60 |
| **Summary of Subject Eligibility Criteria** | Inclusion criteria:   1. Male or female participants aged ≥18 and < 70 years at time of enrollment. 2. Life expectancy > 12 weeks. 3. Participants must have a confirmed diagnosis of multiple myeloma. 4. Refractory multiple myeloma patients 5. Relapsed multiple myeloma patients 6. ALT/AST<3 × institutional upper limit of normal. 7. Total bilirubin ≤2.0mg/dl 8. Performance status (Karnofsky≥50%) 9. No serious liver, kidney, heart and other diseases at the time of enrollment 10. Relapse after auto- or allo- hematopoietic stem cell transplantation (HSCT) 11. Not eligible for HSCT or abandoned HSCT because physical condition is not allowed 12. Voluntarily participated in the trial of CAR-T for the treatment of multiple myeloma 13. Blood was collected intravenously and no other contraindications for aphresis. 14. Informed Consent/Assent: All subjects have the ability to understand and the willingness to sign a written informed consent. |
|  | Exclusion Criteria:   1. Pregnant or lactating women, or female with a pregnancy plan within six months 2. Infectious diseases (eg HIV, active tuberculosis, etc.) 3. Active hepatitis B or hepatitis C 4. Evaluation screening showed that the transfected lymphocytes was less than 10% or proliferation< 5 times under the co-stimulation of CD3/CD28 5. Abnormal vital signs and failure to cooperate with the examination 6. Any mental or psychological illnesses that could, in the investigator's opinion, potentially interfere with the completion of treatment 7. Highly allergic constitution or history of severe allergies, especially to IL-2 8. Systemic or severe local infections require anti-infective treatment 9. Evidence of current uncontrolled dysfunction of heart, lung, brain, kidney and other important organs 10. Active and severe auto-immune disease 11. Other conditions that are not eligible for the trial in the judgment of the principal investigator |
| **Study Product, Dose,**  **Route** | BCMA CAR-T cells and CD19 CAR-T cells, single or fractional dose intravenous injection (total dose of BCMA CAR-T cells and CD19 CART cells was 1× 10^6^ cells/kg body weight, respectively) |
| **Objectives** | The primary objective is to determine the safety and efficacy of sequential infusion of anti-BCMA CAR-T and anti-CD19 CAR-T for relapsed and refractory multiple myeloma  Primary outcome measures：overall remission rate (ORR), complete remission rate (CR), partial remission rate (PR), progression free survial  Secondary outcome measures：safety |
|  | Safety measures  The evaluation of safety will be measured by an assessment of the incidence of treatment-emergent adverse events for each patient in the study. |
| **Statistical**  **Methodology** | The statistical analysis will be primarily descriptive in keeping with the exploratory nature of the study. Descriptive statistics will be applied to determine the persistence and trafficking of the CAR-T to blood. All adverse events will be described and exact 95% confidence intervals will be produced for adverse event rates, both overall and within major categories. Analysis of other secondary endpoints may include summary statistics such as means and standard deviations or Kaplan-Meier curves for survival information. |
| **Study duration** | 4 years |

**1. Background**

Multiple myeloma (MM) is a disease defined by the accumulation of clonal bone marrow plasma cells and development of clinical complications including hypercalcemia, renal insufficiency, symptomatic anemia, destructive lytic bone lesions, and susceptibility to infections. In the past 30 years, the treatment of MM has been developed. From early cyclophosphamide and melphalan to combined chemotherapy in the 1980s, the treatment efficacy of MM has been significantly improved. In recent years, hematopoietic stem cell transplantation (HSCT)^1-2^, protease inhibitor^3^, immunomodulator^4^, Daratumab^5-6^and other applications^7-8^ have further improved the prognosis of MM patients. However, MM is still an incurable disease, especially the prognosis of relapsed and refractory(R/R) patients is still poor^9^. It is necessary to explore more effective treatment methods to improve the prognosis and survival time of R/R MM.

In recent years, immunotherapy has become a focus in cancer therapy^10-14^. Genetically engineered T cells carrying specific antigen binding sequences can specifically recognizeand kill tumor cells in a non-MHC dependent manner^14-16^. T cells modified with chimeric antigen receptors (CARs) specific to MM cell antigen have also been developed and achieved promising response, which has become a new treatment approach for R/R MM^17-20^.

CD19 is the surface marker of B lymphocyte. Almost all B lymphocytic tumors express CD19. Several reports have identified in MM patients a minor component of the multiple myeloma expresses CD19 that is related to drug-resistant, disease-propagating properties ^17, 21^. The treatment of R/R MM with CD19 CAR-T cells (CTL019) has entered Phase I clinical trial (NCT02135406, Pennsylvania, USA). After treated with CTL019 cells followed by malfuran and autologous hematopoietic stem cell transplantation (ASCT), the concentration of monoclonal immunoglobulin (M protein), IL-6 and the expression of immunoglobulin heavy chain gene decreased. 6 of 10 patients in the clinical trial maintained complete remission (CR). The main adverse reactions of CTL019 were grade 1 cytokine release syndrome (CRS) and ASCT-induced enterocolitis^17^.

BCMA is mainly expressed by B cells, normal plasma cells, malignant plasma cells and plasmacytoid dendritic cells, but not by primary B cells, memory B cells, normal hematopoietic stem cells and other non-hematopoietic cells^19, 22^.BCMA belongs to the TNF receptor superfamily, which combines B cell activating factor (BAFF) and a proliferation inducing ligand (APRIL) to promote the growth of MM cells and the adhesion of bone marrow stromal cells^23^. BCMA antibody can kill both MM cell lines and primary MM cells^24^.The number of B cells in BCMA deficient mice is normal, but the function of B cells is inhibited^25.^These data suggest that BCMA is suitable as a target for MM therapy and does not significantly affect the function of normal B cells. The scFv of BCMA-CAR-T cells containing C12A3.2 or C111D5.3 target BCMA-1 and BCMA-2, respectively and CAR-T showed obviously reactive proliferation and killing activity when co-cultured with BCMA-positive MM cells^19^. BCMA CAR-T cells can recognize and kill MM cells of patients, and play an anti-tumor role through perforin pathway in MM murine model. In December 2016, Bluebird Company published the phase I clinical data of BCMA CAR-T in the treatment of refractory and relapsed (R/R) MM. Nine patients were enrolled in the study and received three different doses of CART, e.g. 5.0 x 10^7^(dose 1), 15.0 x 10^7^ (dose 2) and 45.0 x 10^7^(dose 3).The results showed that the overall response rate (ORR) was 78% and 100% in the dose 2 and dose 3 groups, respectively. Two patients met CR criteria at 4- and 6-months follow-up. In dose 2 and dose 3 groups, MM cells were not detected in the bone marrow after 14 days of treatment. In dose 1 group, ORR was 33%. At present, many research institutes around the world are carrying out research on BCMA CAR-T, CD19 CAR-T and other CAR-T cells for the treatment of MM. Preliminary data show that CAR-T can effectively treat R/R MM patients. This study is to determine the safety and efficacy of anti-BCMA CAR-T and anti-CD19 CAR-T in the treatment of R/R multiple myeloma patients. This study may provide data for the new treatment of R/R MM.

**2. Previous research**

**2.1 Construction of Lentivirus Expression Vector for Chimeric Antigen Receptor**

Chimeric antigen receptors targeting CD19 and BCMA were synthesized and subcloned into lentivirus expression vector Lenti-EF1a-puro. The expression vectors targeting CD19 lentivirus are as follows (Figure1):


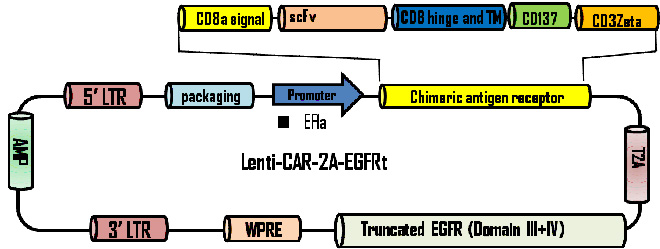


Figure1 Structure of a targeted CD19 lentiviral expression vector

The elements in the schematic diagram are summarized as follows: **EF1a Pro:** This promoter can stably and continuously activate the expression of downstream CAR in T lymphocytes, while CMV promoters are silenced in T cells; **Signal peptide:** A signal peptide that directs the transport of synthesized proteins to cell membranes. **ScFv:** It is composed of single-chain antibodies that can specifically recognize tumor-associated antigens. Heavy-chain and light-chain variable regions are linked by (G4S) 3-peptide fragments. The scFv used in humanizedCD19-CAR-T removes the heterologous protein sequence, while retaining the affinity and specificity of the parent mouse antigen, reducing the possibility of HAMA reaction to the lowest level and effectively prolonging CAR-T cells persistence in vivo; **4-1BB:**4-1BB intracellular functional domain provides co-stimulatory signal for the activation of CAR-T cells; **CD3Zeta:** one of the signals of activation of T cells, can stimulate the proliferation of CAR-T cells after activation. **T2A:** In eukaryotic cells, self-shearing effect can be formed. CAR and EGFRt are expressed as fusion proteins first, and then self-cleaved to form two independent proteins. **EGFRt:** The domain III and domain IV of wild-type EGFR were intercepted. The truncated EGFRt had no intracellular signal and did not transmit other signals to T cells. At the same time, the two domains were the recognition epitopes of cetuximab. After expressing the epitopes on T cells, they could be used as screening markers for CAR-T and a safety switch for clinical research.

**2.2 Manufacturing of clinical-grade self-inactivating lentiviral vectors**

The packaging plasmid required for the production of lentivirus and the chimeric antigen receptor lentiviral expression vector are prepared using an endotoxin-free plasmid extraction kit. 293T cells were cultured in serum-free medium in GMP laboratory, and then co-transfected with liposome. After 72 hours, cell precipitation was removed by centrifugation, supernatant was collected and impurities were removed by positive pressure filtration. Lentiviruses were concentrated by ultrafiltration, and the titers of lentiviruses were determined.

**Product testing:**

Lentivirus titer determination: qPCR, titer >1x10^8^ TU/mL

Mycoplasma detection: PCR, Negative

Bacteria detection: Negative

Fungus detection: Negative

Endotoxin test:<0.5 EU/ml

Detection of replicative recombinant virus: PCR, Negative

**2.3 Manufacture of CD19 CAR-T cells**

PBMC was isolated from peripheral blood of patients and CD3+ T cells were separated by magnetic beads. After activation, T cells were infected with recombinant lentivirus CD19, and CAR-T cells were expanded. The expression of EGFRt on the surface of recombinant CAR-T cells was detected by flow cytometry (Figure2).


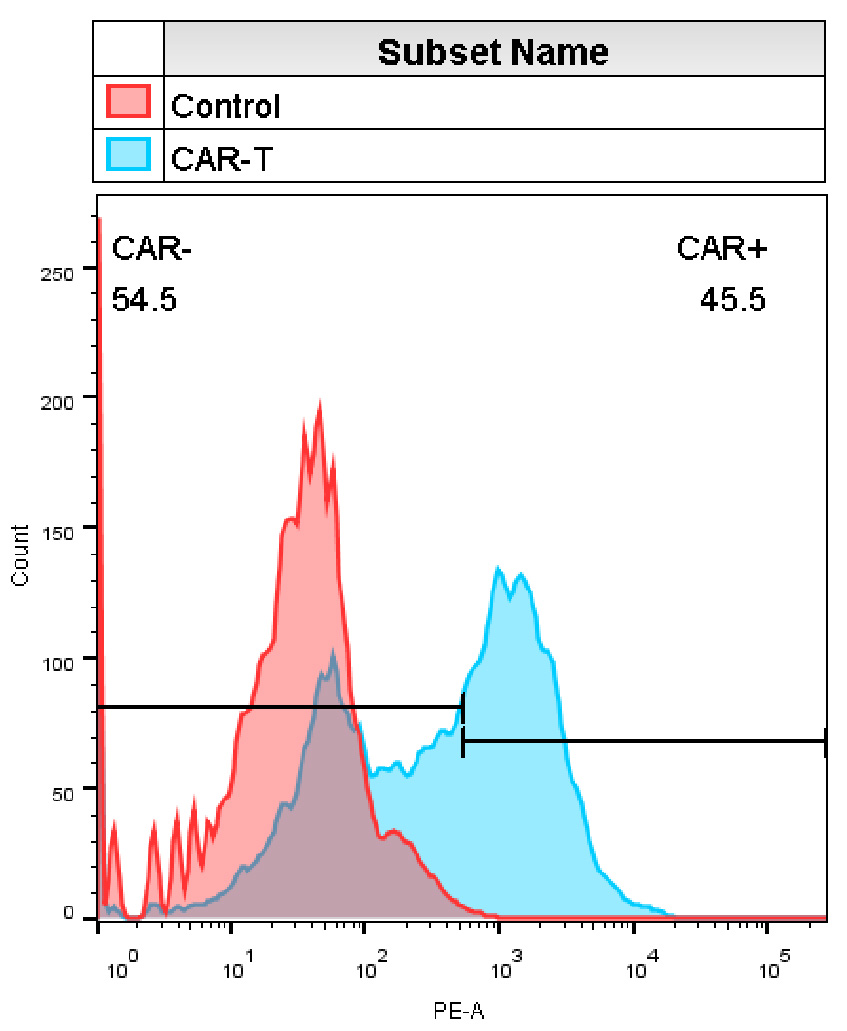


Figure2 CD19 CAR-T cells were identified by flow cytometry

**2.4 Anti-tumor activity of CAR-T cells in vitro**

Using Raji and CHO-K1 cells which express CD19 as target cells and recombinant T cells expressing CD19-CAR as effector cells, a co-culture system of CAR-T cells and targeted tumor cells were established according to different E: T ratios (Figure3). LDH and cytokines released by lysed tumor cells and activated CAR-T cells respectively in supernatant of culture medium were detected to reflect the killing and activating ability of CAR-T cells in vitro (Figure4). At the same time, a control system of co-culture of T cells and tumor cells without transduction was established.


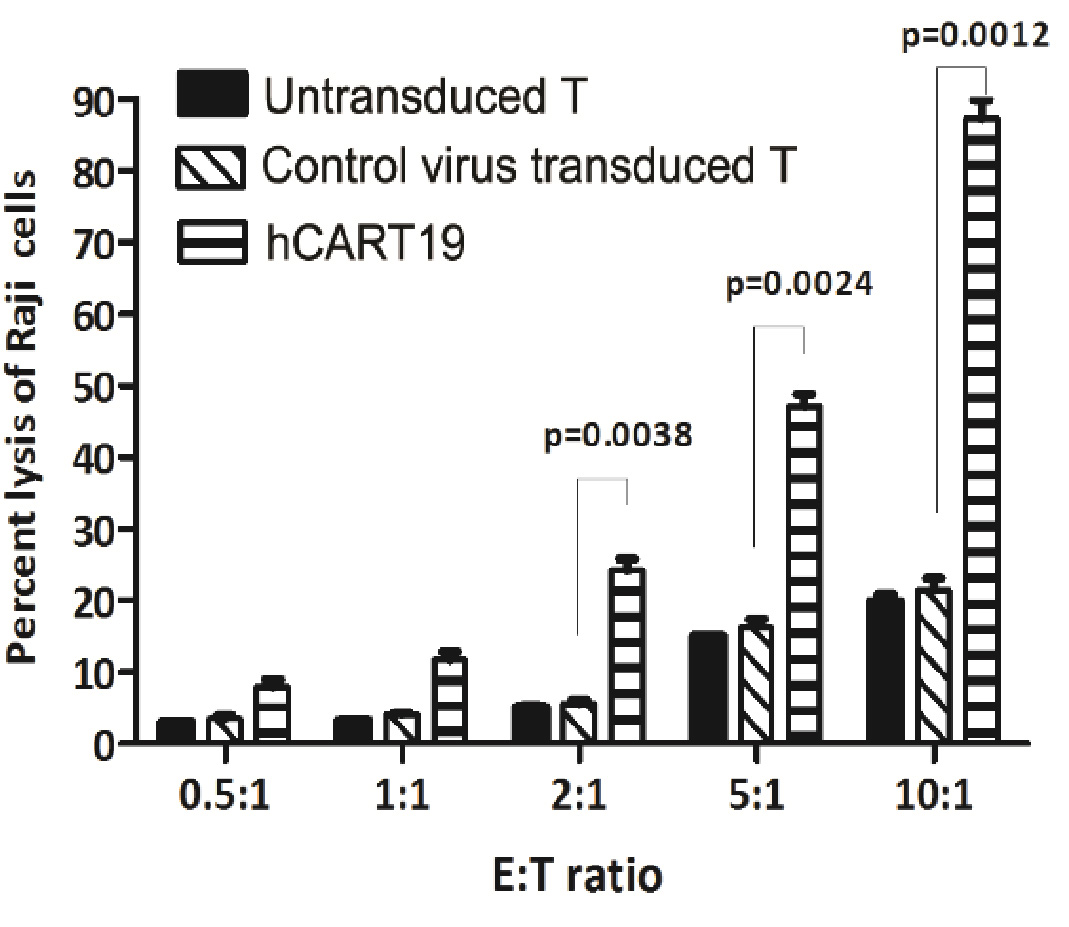


Figure3 Humanized CD19CAR-T cells kill tumor cells


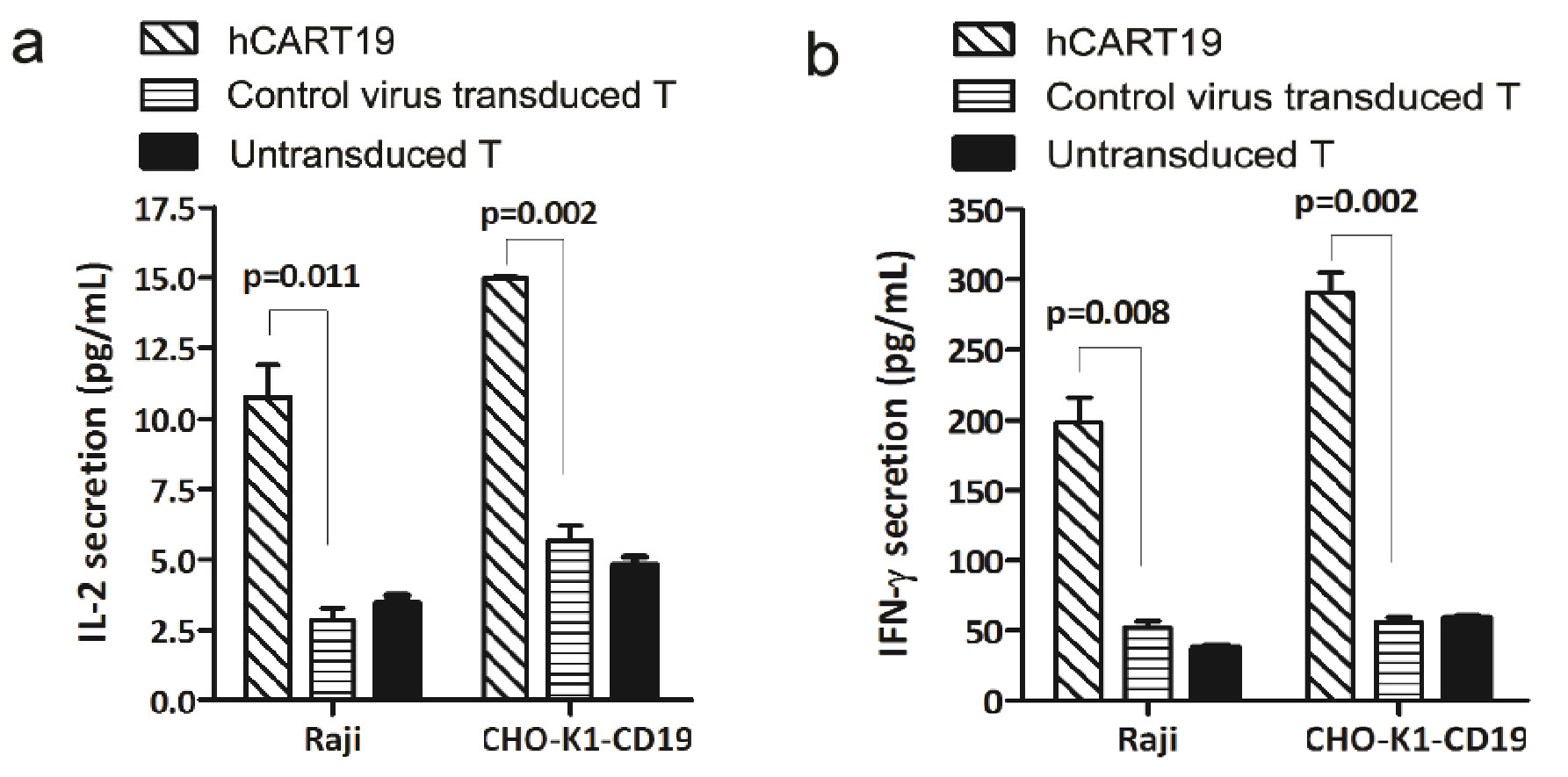


Figure4 panel a shows the release of IL-2 in the co-culture of CAR-T with target cells; panel b shows the release of IFN-γin the co-culture of CAR-T with target cells.

**2.5 Anti-tumor activity of CAR-T cells in vivo**

The recombinant Raji cells expressing luciferase were constructed. During the first two days, 5×10^5^ and 5×10^6^ CAR-T cells were infused through the tail vein. At the indicated time point, the mice were briefly anesthetized and injected with Luciferin for in vivo imaging (Figure5).


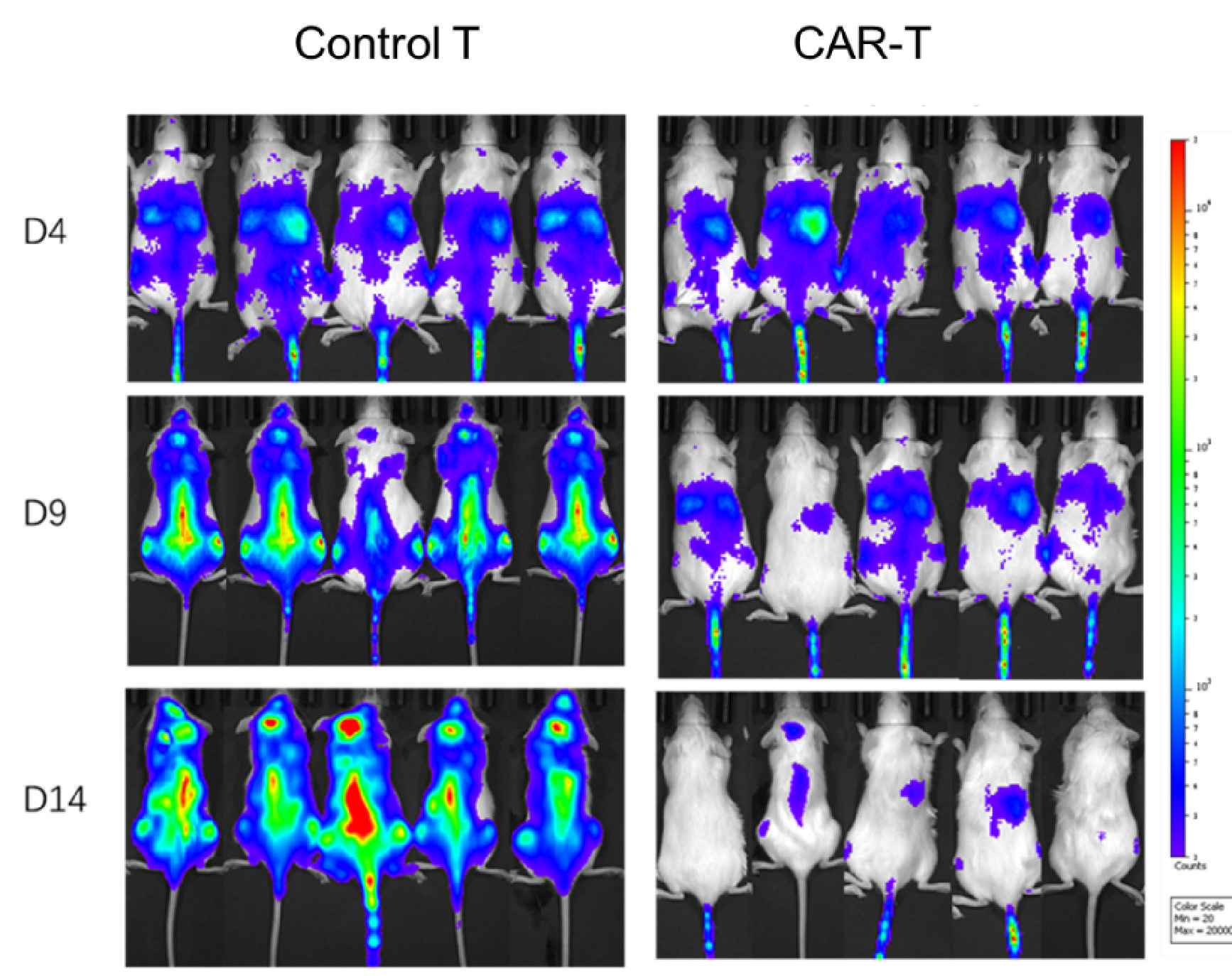


Figure5 In vivo imaging of mice after infusion of Raji cells

**2.6 Manufacturing BCMA CAR-T**

PBMC was isolated from peripheral blood of patients and CD3+ T cells were separated by magnetic beads. After activation, T cells were infected withrecombinant lentivirus BCMA, and CAR-T cells were expanded. The expression of EGFRt on the surface of recombinant CAR-T cells was detected by FACS (Figure6).


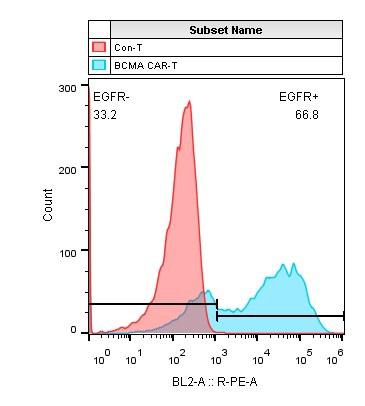
Figure 6 BCMA CAR-T cells were identified by flow cytometry

**2.7 CAR-T anti-tumor activity in vitro**

Using U266 and CHO-K1 cells expressing BCMA as target cells and recombinant T cells expressing BCMA-CAR as effector cells, a co-culture system of CAR-T cells and targeted tumor cells was established according to different E:T ratios (figure7). LDH released by lysed tumor cells and cytokines released by activated CAR-T cells in supernatant of culture medium were detected to reflect the killing and activating ability of CAR-T cells in vitro (Figure8). At the same time, a control system of co-culture of T cells and tumor cells without transduction was established.


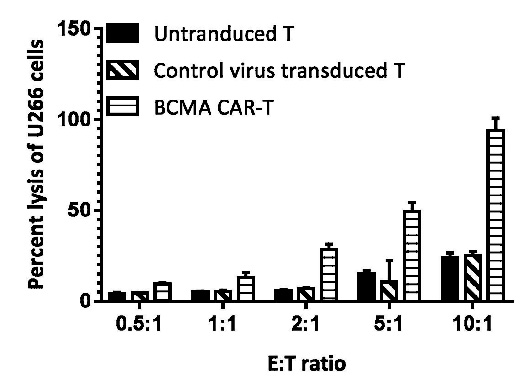


Figure7 BCMACAR-T cells kill tumor cells


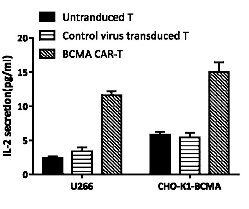

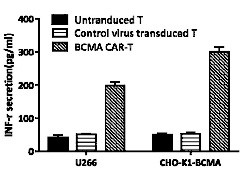


Figure8 the panels show released IL-2 and IFN-γ when CAR-T is co-cultured with target cells

**2.8 BCMA CAR-T anti-tumor**

The recombinant U66 cells expressing luciferase were constructed. During the first two days, 5×10^5^ and 5×10^6^ CAR-T cells were infused through the tail vein. At the indicated time points, the mice were briefly anesthetized and injected with Luciferin for in vivo imaging.

**
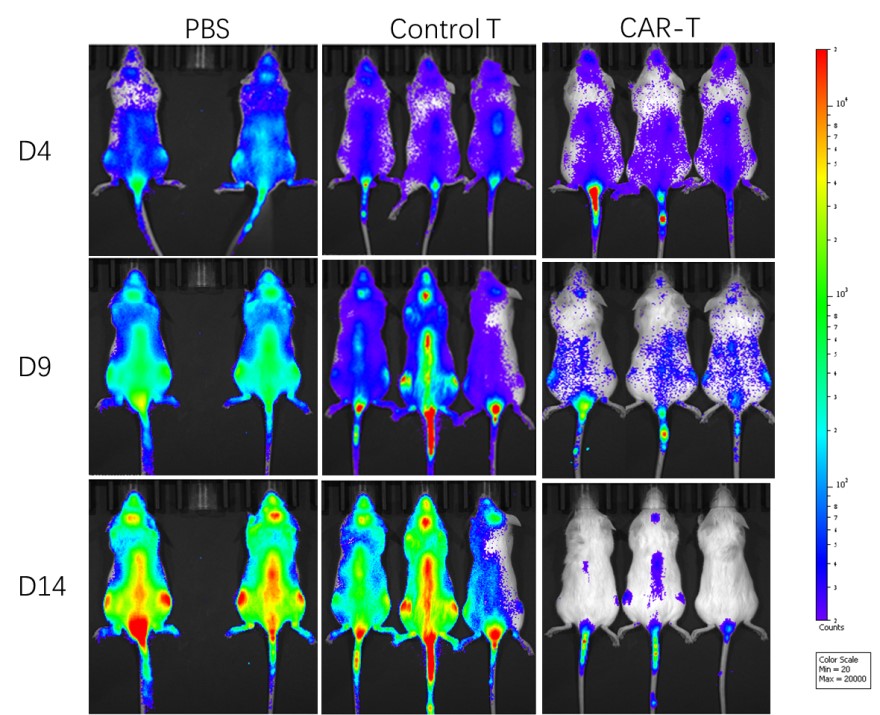
**

Figure9 U266 cells show superior tumor burden control in vivo

1. **Objective**

This study is a single-center, open, single-arm clinical study. we attempted to treat patients with R/R MM through combination of humanized anti-CD19 CAR-T and anti-BCMA CAR-T, to observe hematological toxicity, Overall survival (OS) and Event-free survival (EFS).

1. **Study Design, Principles and Test Procedures**

4.1 Study design

This is a single-center, open-label pilot study to assess the safety and feasibility of sequential infusion of anti-BCMA CAR-T and anti-CD19 CAR-T relapsed and refractory multiple myeloma.

4.2 Sample

Number of sample：60

4.3 Study duration: 4 years

4.4 Enrollment of patients

Inclusion criteria:

This is a single-center, open-label, single-arm clinical study in which all enrolled patients with an age over 18 but less than 70 years met the International Myeloma Working Group (IMWG) diagnostic criteria^26^. Patients who were eligible for this study were confirm to be MM through histology, immunology, imaging examination and monoclonal immunoglobulin (or light chain) and had been staged according to the Durie-Salmon (DS) staging system. All patients have been treated with alkylators, proteasome inhibitors or autologous hematopoietic stem cell transplantation, and who were refractory to the last line of treatment(response less than partial response (PR) after most recent myeloma therapy or progressive disease (PD) within 60 days after most recent myeloma therapy.)

1. Male or female participants aged ≥18 and < 70 years at time of enrollment.
2. Life expectancy > 12 weeks.
3. Participants must have a confirmed diagnosis of multiple myeloma.
4. Refractory multiple myeloma patients
5. Relapsed multiple myeloma patients
6. ALT/AST<3 × institutional upper limit of normal.
7. Total bilirubin ≤2.0mg/dl
8. Performance status (Karnofsky≥50%)
9. No serious liver, kidney, heart and other diseases indicated at the time of enrollment
10. Relapse after auto- or allo- hematopoietic stem cell transplantation (HSCT)
11. Not eligible for HSCT or abandoned HSCT because physical condition is not allowed
12. Voluntarily participated in the trial of CAR-T for the treatment of multiple myeloma
13. Informed Consent/Assent: All subjects must have the ability to understand and the willingness to sign a written informed consent.

Exclusion Criteria:

1. Pregnant or lactating women, or female with a pregnancy plan within six months
2. Infectious diseases (eg HIV, active tuberculosis, etc.)
3. Active hepatitis B or hepatitis C
4. Evaluation screening showed that the transfected lymphocytes was less than 10% or proliferation< 5 times under the co-stimulation of CD3/CD28
5. Abnormal vital signs and failure to cooperate with the examination
6. Any mental or psychological illnesses that could, in the investigator's opinion, potentially interfere with the completion of treatment
7. Highly allergic constitution or history of severe allergies, especially to IL-2
8. Systemic or severe local infections require anti-infective treatment
9. Evidence of current uncontrolled dysfunction of heart, lung, brain, kidney and other important organs
10. Active and severe auto-immune disease
11. Other conditions that are not eligible for the trial in the judgment of the principal investigator

Suspension criteria

Subjects who did not complete the protocol were considered to stop the trial as soon as possible. Causes of premature termination (e.g., voluntary evacuation, toxic side effects, and deaths) must be documented in case reports. The research evaluation will be completed at the end of the suspension. Potential reasons for premature suspension include：

1. The follow-up of this subject failed.
2. The main researchers believe that the disease is too serious to continue.
3. Patients do not comply with the treatment and clinical agreement of the study;
4. Pregnancy;
5. Voluntary withdrawal. Subjects may withdraw from research projects at any time by changing their will.
6. The significant and rapid development of malignant tumors and metastasis of central nervous system need to choose other methods, including radiotherapy or surgical treatment, but not limited to these two methods.
7. Technical difficulties encountered in the process of T cell gene modification and amplification hinder the production of clinical cell doses that meet the quality control standards.
8. In addition, if the number of patients with serious adverse events related to cell infusion exceeds 40% of the patients in the enrolled group, the researcher will suspend the study and conduct the corresponding demonstration to decide whether to continue the project.

**4.5 Study Methodology**

This study consists of 4 steps: Recruitment and Screening of Subjects; Preparation of CAR-T infusion (including leukocyte apheresis, CAR-T cell preparation and chemotherapy); Patients were treated with CAR-T cells; Post-treatment assessment and follow-up.

4.5.1 **Recruitment and Screening of Subjects**

4.5.1.1 First, evaluate the patient's medical history to see if the disease meets the indication requirements; then explain the treatment process and risks, and patient sign an informed consent form. Blood samples were taken and tested for HIV and sent to the CAR-T production department for T cell production feasibility assessment. About 1-2 weeks, proliferation and transduction efficiency of the patient's T cells will be assessed by production departmentin vitro to confirm whether his/her T cells are suitable for large-scale CAR-T cell production.

**4.5.1.2 Patients recruitment and data collection**

The patients who passed the previous stage were examined for diseases and a series of routine examinations. See the following examination items for details. The results of the examination items were collected. According to the Inclusion criteria and exclusion criteria, the suitability of CAR-T cell therapy was confirmed.

Screen tests (the following tests must be completed within 4 weeks after enrollment):

1. Complete history and physical examination.
2. Assessment of cardiac function by electrocardiogram and MUGA scans or cardiac ECHO
3. Pulse Oxygen Saturation Assessment of Pulmonary Function.
4. Na, K, BUN, creatinine, Cl, bilirubin, Ca for whole blood cell count, PO4, CO2, LDH, ALT, AST, uric acid, haptoglobin, direct and indirect Coombs tests, beta-2 microglobulin, SPEP, HCG (for premenopausal women), immunoglobulin levels (IgG, IgM and IgA), free light chain, PT/APTT and INR.
5. Bone marrow aspiration, cytogenetics.
6. To quantify the number of T cells and the ratio of CD4:CD8 of Peripheral blood by flow cytometry.
7. Detection of HIV, hepatitis B (HepBS Ab, Hep BS Ag, Hep Be Ab, Hep Be Ag and HepBc Ab), and hepatitis C (Hep C Ab).

**4.5.2 Leukocyte apheresis and CAR-T cell production**

4.5.2.1 Lymphocytes were isolated from peripheral blood of enrolled patients using a blood cell separator and T lymphocytes were sorted using immunomagnetic beads.

**4.5.2.2 Humanized anti-CD9 CAR-T cell production**

**4.5.2.2.1 Design of humanized anti-CD19 antibody**

The variable regions of the mouse anti-human CD19 antibody (FMC63) were humanized through complementarity-determining region (CDR) grafting and structure-based back mutations to replace the non-human frameworks. Humanized candidate heavy and light chains were randomly aligned with a GGGGSGGGGSGGGGS linker. All humanized candidates were submitted to General Biosy stems for gene synthesis and subsequently subcloned into the pFUSE-hIgG1-FC2 vector. All recombinant single chain Fv-expressing vectors were confirmed by Sanger sequencing.

**4.5.2.2.2 Expression and purification of the recombinant scFv antibody**

All humanized single-chain variable fragment (scFv) antibodies were expressed by transient transfection into CHO-S cells (Thermo Fisher Scientific, Waltham, USA) and purified by a Protein A column. Briefly, 100 mL of CHO-S cells was prepared in Freestyle CHO medium. Next, 100 μg of scFv expression vector and 200 μL of 100 μ Mpolyethyleneimine (PEI) stock solution were diluted in 5 mL of PBS buffer, followed by a 30-min incubation after thorough mixing. After the addition of the DNA/PEI complex, the CHO-S cells were maintained on an orbital shaker at 130 rpm in a 37°C incubator. After 6 hours, 100 mL fresh culture medium was supplemented and the cells were continuously cultured for 6 days. After collection of the supernatant, the scFv antibody was purified by a Protein A column. The chimeric scFv antibody was simultaneously expressed as a control.

**4.5.2.2.3 Flow cytometry (FACS) analysis of humanized scFv antibody binding to CD19**

Recombinant CHO-K1 cells expressing heterogeneous human CD19 were previously established in our department (Cao J et al., Am J Hematol 2018) and herein served as the positive control cells to determine the binding affinity of humanized scFv antibodies. Approximately 1 × 10^6^ cells were incubated with 10 μg of humanized scFv antibody for 30 min at room temperature. Then, PE-conjugated anti-human IgG (BD Bioscience, San Diego, USA) was added. After three washes, the CHO-K1-CD19 cells were resuspended in 500 μL of PBS buffer, followed by FACS analysis. All FACS data were processed using the FlowJo software.

**4.5.2.2.4 Generation of the lentiviral chimeric antigen receptor**

The humanized scFv antibody variable fragment was inserted into tandem with the human CD8 transmembrane, CD8 hinge, 4-1BB costimulatory domain, CD3ζ intracellular region and T2A-EGFRt sequence. The full chimeric antigen receptor was then subcloned into the lentiviral vector Lenti-EF1a. The lentiviral supernatant was generated by transient co-transfection of 293T cells with PEI and the Lentipackaging mix (iCarTab Co., Su Zhou, China). The left virus-containing supernatant was concentrated through ultracentrifugation at 50,000 x g for 90 min at 4°C, and the lentiviral pellet was resuspended in PBS buffer and stored at -80°C.

**4.5.2.2.5 Generation of hCART19s**

Enriched monocytes from healthy donors or patients were further isolated via density gradient centrifugation (Lymphoprep, Axis Shield, Norway) at 800 g without break for 20 min at room temperature. The interface, peripheral blood mononuclear cell (PBMC) layer, was collected and washed with 0.9% Saline. CD3+ T cell purification and activation were conducted with magnetic Dynabeads (Thermo Fisher Scientific) following the manufacturer’s instructions. At 24 hours after activation, T cells were transduced with CD19 lentivirus (MOI=5). Briefly, 3×10^6^ T cells were mixed with 100 μL of lentivirus (108 TU/mL) and centrifuged at 800 g for 60 min at 30°C. After centrifugation, the cell culture flask was returned to a 37°C and 5% CO2 incubator. The transduced T cells were continuously cultured for 5-10 days prior to the subsequent assay.

**4.5.2.3 Anti-BCMA CAR-T Cell Production**

Anti-BCMA single-chain variable region is derived from mouse anti-human BCMA monoclonal antibody, 4-1BB is a costimulatory molecule, and CD3ζ is a T cell activation domain. The process is same as the process of humanized anti-CD9 CAR-T.

4.5.2.4 Patients preparation

Recruit patients with refractory and recurrent multiple myeloma who volunteer to participate in clinical research, and fully inform the potential benefits and inconveniences of clinical trials. After signing the informed consent form, physical examination and laboratory examination were carried out, and patients who met the selection criteria were screened according to the examination report.

4.5.2.5 **Chemotherapy**

The aim of chemotherapy is to reduce the burden of tumors and deplete the endogenous lymphocytes, so as to facilitate the proliferation of reinfused CAR-T cells. All patients were pretreated with FC regimen, fludarabine: 30mg/m^2^x 3 days, cyclophosphamide: 750mg/m^2^×1 day. Antiemetic and symptomatic treatment can be given in the course of chemotherapy. In addition to contra-indications, general treatment is the same as other chemotherapy. If neutrophils are less than 1.0 ×10^9^/L after chemotherapy, granulocyte colony stimulating factor should be applied to neutrophils more than 1.5×10^9^/L. Antibiotics can be given to prevent infection if patients have neutrophil deficiency.

**4.5.2.6 Assessment post chemotherapy**

After chemotherapy, tumor burden should be re-evaluated. Screen tests should include imaging diagnosis, physical examination, blood laboratory testing, bone marrow MRD assessment, and chemotherapy side effects assessment.

**4.5.3 CAR-T cells infusion**

CAR-T cells were infused two days after the end of chemotherapy. The CART-19 cells are cryopreserved in infusible cryomedia and will be administered as a single dose. The entire return process should be within 10-15 minutes. During the entire infusion process, the patient's vital signs should be closely monitored. The oxygen saturation test should be performed 15 minutes before the infusion, at the end of the infusion, 15 minutes after the infusion, and until the patient's condition is stable. 30 to 60 minutes before infusion of CAR-T cells, patients were given 325 to 650 mg of acetaminophen to prevent infusion-related reactions. If fever occurs on the day of transfusion of CAR-T cells and lasts less than 24 hours without any other toxicity, it is attributed to the transfusion of T-cell response.

**4.5.4** Post-treatment assessment and follow-up

**4.5.4.1 Proliferation of CAR-T cells**

To assess the in vivo amplification of CAR-T cells, we designed primers for CD19 CARs and BCMA CARs and probes with fluorophores to detect the proliferation of humanized CD19 CAR-T and BCMA CAR-T cells in peripheral blood by quantitative PCR and detailed process were included in the supplementary materials. T lymphocytes and B lymphocytes in peripheral blood was detected according to the procedure with details in the Appendix 3.

**4.5.4.2 Concomitant medication**

All kinds of chemotherapeutic drugs (such as aggravation or recurrence) are not allowed in patients with CAR-T cell transfusion. The complications of patients with CAR-T cell infusion can be treated symptomatically, unless severe cytokine release syndrome is difficult to control with other drugs, tropizumab or glucocorticoid can be chosen.

**4.5.4.3 Observation schedule**

Observation indicators and examination time were carried out according to the requirements of follow-up time.

**4.5.4.4 Follow-up**

Subjects were assessed according to the plan (weekly assessment within one-month, monthly assessment within six months, and subsequent assessment every three months). Therapeutic effect was assessed every four weeks and every three months after six months. After a close follow-up of 6 months, the subjects will undergo a quarterly medical history assessment, physical examination and blood examination for two years. After this assessment, participants will enter a five-year annual telephone follow-up and questionnaire study to assess long-term health problems such as recurrence of malignant tumors.

1. **Clinical efficacy evaluation**

The response was assessed according to the IMWGcriteria^27^with details in the Appendix（Appendix 4,5）. Re-evaluation of the response was performed two weeks, 1 month, 2 months, 3 months, 6 months, and one year after CAR-T infusion. The evaluation included: The number of plasma cells in the bone marrow will be determined by the use of morphology and flow cytometry; To monitor disease progression and residual disease levels; serum paraprotein, serum immunoglobulin level measured by electrophoresis, immunofixation and serum free light chains. MRD was detected according to the standard proposed by Dongen et al. ^28^ with details in the（Appendix 6）. In patients with extramedullary disease, the assessment included imaging techniques and physical examination. Patients should be reassessed and given salvage therapy under the condition of disease progression or relapse at any time.

**Effectiveness evaluation**

1. Survival: overall survival (OS), disease-free survival (DFS), progression-free survival (PFS)
2. Quality of life (QOL) score of patients
3. Evaluation of anti-tumor response: overall response rate (ORR), strict complete remission (sCR), complete remission (CR), partial remission (PR), disease stability (SD), disease progression (PD)
4. Baseline assessment of tumor imaging (18F-FDG PET imaging)
5. CAR-T assessment: Flow cytometry and quantitative PCR were used to detect periodically the presence of CAR-T cells in peripheral blood and bone marrow.

**Safety evaluation**

1. Detection of CRS-related factors: IL-6, IL-10, TNF, IFNγ etc.
2. Detection of various laboratory items: blood routine, coagulation function, blood piece observation, liver function, kidney function, electrolyte, blood sugar, myocardial enzymes, T cell subsets, immunoglobulin, etc.
3. Observation of adverse events and serious adverse events
4. **Project risk**

Safety assessment included all patients receiving at least one dose of cells. According to the treatment group, we summarized the treatment-related adverse events, severe adverse events, treatment-related serious adverse events, safety laboratory parameters, and classified the adverse events according to NCI CTCAE version 5.0^29^ and the CRS evaluation criteria proposed by Lee et al^30^(Appendix 7).

# 6.1 Adverse event (AE)

# Adverse events (AEs) refer to any adverse medical manifestations, all abnormal findings, subjective and objective symptoms, complications and accidents that occur during cell infusion. Events may arise from the use of cells or from accidental or intentional overdose, poisoning, abuse or withdrawal of drugs. Any aggravation of an existing condition or disease is considered an adverse event. Abnormal laboratory results and changes in ECG are considered adverse events only when they lead to discontinuation of treatment, need therapeutic intervention, or what researchers consider to be AEs.

# 6.2 Observation of adverse events

**6.2.1 Clinical adverse events**

All participants were carefully observed for any adverse events during the study, and their clinical manifestations, severity, occurrence time, duration, treatment methods and prognosis were recorded in time. The correlation between adverse events and experiments was also determined. All adverse events were followed up until relapses of disease.

**6.2.2 Abnormal laboratory test**

# Those with abnormal results should be closely followed up until they returned to normal, and the correlation between the abnormal results and the treatment should be determined.

# 6.3 Severity of AE

**Table 1-Severity of AE**

| **mild** | Transient and tolerate |
| --- | --- |
| **moderate** | Uncomfortable, normal activities are affected |
| **Severe** | Normal activities are severely limited, may cause disability and life threatening |
| **Life threatening** | Life threatening immediately |
| **dead** | Correlated with AEs |

# 6.4 Criteria for the relationship between AEs and experimental treatment

**6.4.1 Criteria for evaluating adverse events (including symptoms, signs, test indicators)**

1) The time of occurrence of adverse events coincided with the time of cell therapy.

2) AEs are associated with known adverse reactions to cell therapy

3) AEs cannot be explained by other reasons.

4) AEs disappeared after cell arrest therapy

5) Reproduction of AEs after cell therapy

**Table 2 - Modified Karch and Lasagna (1975) versions describe the relationship between adverse events and the drug being tested**

| **Definite** | A response occurs when the drug is taken for a short period of time, or when the drug accumulates a certain level in the body fluid or tissue, triggering a known or expected response pattern of the suspected drug; and the response improves precisely after withdrawal or reduction of the dose, but the response reappears after repeated use. |
| --- | --- |
| **Credible** | A response occurs when the drug is taken for a short period of time or when the drug accumulates a certain level in the body fluid or tissue, triggering a known or expected response pattern of the suspected drug; and the response will definitely improve after withdrawal or reduction of the dose, but it cannot be reasonably explained by the clinical condition known to the subject. |
| **Possible** | A reaction occurs in a short time after taking the drug under test because the suspected drug has a known or expected response pattern, but the reaction can also be easily triggered by other factors. |
| **inaccessible** | An association cannot be established by evaluation. |
| **Unrelated** | A reaction in which there is sufficient information to indicate that the cause of the disease is not related to the drug being tested. |

**6.4.2 Adverse reaction assessment**

- definite correlation: meeting the above criteria 1, 2, 3, 4 and 5
- Possibly correlated: meeting the above criteria 1, 2, 3 and 4
- inaccessible: at the same time meet the above standards 1 and 2
- may unrelated: meeting the above-mentioned Article 1 criteria
- Definite unrelated: none of the five criteria mentioned above is in conformity with it.

**Table 3-assessment of AE**

| results | indicators | | | | |
| --- | --- | --- | --- | --- | --- |
|  | 1 | 2 | 3 | 4 | 5 |
| Definite | + | + | + | + | + |
| Credible | + | + | + | + | ？ |
| Possible | + | + | ± | ± | ？ |
| inaccessible | + | − | ± | ± | ？ |
| Unrelated | + | − | − | − | − |

# 6.5 Record of AEs

**6.5.1 AEs**

All AEs should be recorded in the CRF and the following should be provided as far as possible:

- The nature of adverse events
- The time when adverse events first occurred
- The intensity of adverse events
- Relations with CAR-T therapy
- The duration of adverse events
- Whether the event is one-off or intermittent (theoretically, every adverse event should be reported).

However, some AEs may occur frequently, such as vomiting or diarrhea; if the interval between intermittent events is less than 24 hours, it is more reasonable to record them as an intermittent adverse event.)

- Severe/non-severe

**6.5.2 Severe Adverse Events (SAEs)**

SAE are events that require hospitalization, prolong hospitalization time, impair work ability, endanger life or death, and cause congenital malformations during clinical trials.Any serious adverse event that occurs during the treatment or observation phase, whether or not it is related to the research drug, must be reported by fax to the coordinating organization/its representative within 24 hours of his/her discovery.

Researchers are required to record all serious adverse events in the CRF Serious Adverse Events Page and fax them to the coordinating organization/its representative to report serious adverse events. Researchers should not delay notifying coordinating organizations/their representatives of serious adverse events while waiting for other information to complete all records. The information contained in the initial notice should at least be sufficient to illustrate the following:

- number
- The abbreviation of the subject's name (for reasons of confidentiality, the subject's name should not be transmitted)
- The time and date of the first study drug use
- The time and date of the incident
- A brief description of what happened and the countermeasures taken
- Researchers' opinions on the relationship between events and research drugs

# After the first fax report, the CRF Serious Adverse Events Page should be used to further report the adverse events, detailing the questionable parts of the adverse events. Relevant hospital case records and anatomical reports should be provided as far as possible. Coordination organizations should report SAEs to local regulatory bodies in accordance with local regulations. Researchers must report serious adverse events to regulatory authorities (if applicable) and ethics committees (IRB) in accordance with local laws and regulations.

# 6.6 Follow-up observation of subjects with non-severe and severe adverse events

All subjects with non-severe and severe adverse events, whether or not they are associated with the drug, must be monitored. Until the symptoms subside and the relevant clinical laboratory parameters fall back to baseline, or observed changes are satisfactorily explained, or death, pathological reports should be provided if feasible. All results must be reported in the files.

# 6.7 Adverse Event Collection Time

From the beginning of the study to the end, all non-severe adverse events occurring throughout the study need to be collected, whether they are regular telephone contacts and/or information from research visits or spontaneous reports from recipients.

SAEs were collected from the beginning of CART cell therapy until 3 months after completion or discontinuation of CART cell therapy.

**7. Risk disposal SOP**

The symptoms of cough, low fever, fatigue, muscle pain, shivering, sweating, anorexia, nausea and diarrhea were closely observed after CAR-T cell infusion. Whether the patient has respiratory or heart-related symptoms. The presence of tumor lysis symptoms was closely observed for the first two weeks after injection.The main indicators included serum electrolyte, phosphorus, calcium, uric acid, creatinine and lactate dehydrogenase. The subjects were assessed for toxicity according to the plan. （Weekly assessment for one month, monthly assessment for six months, and subsequent assessment for three months）

**7.1 Hypogammaglobulinemia and B/plasma cell aplasia**

Transient or permanent host B and plasma cell depletion and hypogammaglobulinemia is also a potential risk with CART-19 cells, since normal B cells express CD19. This is expected to resolve if the CART-19 cells are cleared.

**7.1.1 Clinical Indications of Intravenous Infusion of Immunoglobulin**

①IgG< 2g/L;

②IgG 2-5g/L，Patients with antibody deficiencies and frequent bacterial infections.

**7.1.2 Dosage of IVIgG**

0.4g-0.6g/kg/month；

To monitor IgG concentration (after the end of a diagnosis and treatment cycle), ensure that IgG concentration should be close to or higher than the lower limit of IgG concentration in normal people.

**7.2 Cytokine Release Syndrome（CRS）**

Patients treated with CART-19 may experience CRS, development of which has correlated with disease response. Clinical manifestations have included high fevers, fatigue, anorexia, nausea, vomiting, headache, rash, hypotension (occasionally requiring vasopressor support), tachypnea, hypoxia (occasionally requiring ventilator support), delirium and confusion (in several patients), evidence of disseminated intravascular coagulation as well as MAS. The CRS has been effectively abrogated with anti-cytokine directed therapy including Dexamethasone or tocilizumab in most patients.

**7.2.1 Diagnosis**

| **Organ system** | **Symptoms** |
| --- | --- |
| Constitutional | Fever ± rigors, malaise, fatigue, anorexia, myalgias, arthalgias, nausea, vomiting, headache |
| Skin | Rash |
| Gastrointestinal | Nausea, vomiting, diarrhea |
| Respiratory | Tachypnea, hypoxemia |
| Cardiovascular | Tachycardia, widened pulse pressure, hypotension, increased cardiac output (early), potentially diminished cardiac output (late) |
| Coagulation | Elevated D-dimer, hypofibrinogenemia ± bleeding |
| Renal | Azotemia |
| Hepatic | Transaminitis, hyperbilirubinemia |
| Neurologic | Headache, mental status changes, confusion, delirium, word finding difficulty or frank aphasia, hallucinations, tremor, dymetria, altered gait, seizures |

7.2.2 CRS grade system

| **Grade** | **Toxicity** |
| --- | --- |
| Grade1 | Symptoms are not life threatening and require symptomatic treatment only, eg, fever, nausea, fatigue, headache, myalgias, malaise |
| Grade 2 | Symptoms require and respond to moderate intervention |
| Oxygen requirement <40% or |
| Hypotension responsive to fluids or low dose2 of one vasopressor or |
| Grade 2 organ toxicity |
| Grade 3 | Symptoms require and respond to aggressive intervention |
| Oxygen requirement ≥40% or |
| Hypotension requiring high dose**[*](http://www.bloodjournal.org/content/124/2/188?sso-checked=true#fn-2)** or multiple vasopressors or |
| Grade 3 organ toxicity or grade 4 transaminitis |
| Grade 4 | Life-threatening symptoms |
| Requirement for ventilator support or |
| Grade 4 organ toxicity (excluding transaminitis) |
| Grade 5 | Death |


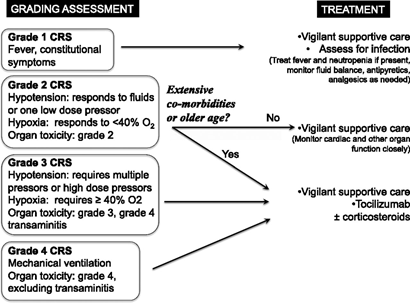
**7.2.3 CRS therapy**

**7.3 Risk of tumor lysis syndrome (TLS) related to cytoreductive chemotherapy or CAR T cells**

The risk of tumor lysis syndrome (TLS) is dependent on the disease burden, but in most cases, this risk will be very low. The burden of CART-19 target cells in MM patients is low compared to the CLL patients previously treated, though MM does rarely express CD19. Patients will be closely monitored both before and after chemotherapy and CART-19 infusion for laboratory evidence of TLS (serum measurements of potassium, uric acid, phosphate, calcium, and creatinine). Subjects will receive hydration, allopurinol (at the discretion of the treating physician), to minimize any toxicity should significant acute tumor lysis begin to occur.

**7.4 MAS/HLH**

Features consistent with MAS/HLH have been observed in patients treated with CART-19, coincident with clinical manifestations of the CRS. MAS appear to be a reaction to immune activation that occurs from the CRS and therefore should be considered a manifestation of CRS. MAS is similar to HLH; it is a rare reaction to immune stimulation by infection, autoimmune diseases or other precipitants, but it is distinct from familial or genetically mediated HLH. There are no definitive diagnostic criteria for MAS, but it is typically diagnosed by meeting HLH diagnostic criteria. Some but not all features of MAS are typically observed with the CRS that develops in CART-19 recipients. The clinical syndrome of MAS is characterized by high grade non-remitting fever, cytopenias affecting at least two of three lineages, and hepatosplenomegaly. It is associated with biochemical abnormalities, such as high circulating levels of serum ferritin, soluble interleukin-2 receptor (sCD25), and triglycerides, and decrease in circulating natural killer cell activity. Other findings include variable degrees of hepatic dysfunction ranging from mild transaminase elevation up to signs of acute liver failure and coagulopathy and DIC. A pathologic feature of MAS is the presence of hemophagocytic CD163+ macrophages (HPC) in bone marrow or lymph-node aspirates. Research correlative studies have shown that IL-6 levels were extraordinarily high during the CRS, prompting us to use an anti-IL6 receptor antibody tocilizumab to treat the CRS/MAS. The majority of patients treated with tocilizumab had rapid (within hours) resolution of dramatic fevers, and continuous improvement in hypotension and hypoxia over hours to several days, and showed improvement in biochemical evidence of CRS and MAS within 48 hours. Adult patients were treated with tocilizumab 4mg/kg or 8 mg/kg.

**8.Regulation**

**8.1 Personnel**

The medical team consists of at least one senior doctor, two attending physicians, one senior nurse with the title of Vice-Senior or above, and two supervisor nurses. Medical staff should be skilled in aseptic operation and first-aid skills. Lent viral construction and cell preparation technicians are healthy and free from infectious diseases. They are skilled in aseptic operation and have more than 3 years’ experience in GMP viral construction and human T cell culture.

**8.2 Environment**

According to the "Code for Construction and Acceptance of Cleanrooms", "Good Manufacturing Practices for Pharmaceutical Production (Revised in 2010)", GMP laboratory that has passed the examination and has an overall Class B local A-level standard. The construction of the lentiviral system and the entire process of CAR-T cell preparation were performed in a GMP laboratory.

**8.3  Apparatus**

Lentivirus construction and CAR-T cell preparation equipment are standardized. All equipment is regularly maintained and calibrated.

**8.4 CAR-T cells preparation**

Before infusion, CAR-T products must pass the following test items: bacteria (e.g. faecalalkalogenic bacteria, Candida albicans, Escherichia coli, Haemophilusinfluenzae, Meningococcus, Pseudomonas aeruginosa, Staphylococcus aureus, Streptococcus pneumoniae and Streptococcus pyogenes A), fungi, mycoplasma, endotoxin, replicated lentivirus, p24, VSV-G nucleus, Acids,HIV gag, mouse antibodies,medium components, carrier packaging cells or plasmid components.

**8.5 Files**

The hospital should keep these original data until 5 years after the termination of the clinical study, including the confirmation of all participants (effective check of different records, such as CRF and hospital original records), informed consent of all original participants, CRF form, detailed records of drug distribution, etc.

**Files and data**

The inspector is responsible for conducting regular on-site inspections to ensure strict compliance and implementation of clinical trial programs. The record table are checked to ensure that the data on CRF are consistent with the original data, no errors and no tampering. All adverse events are recorded in detail, properly handled and tracked until they are properly resolved. SAE and unexpected events are reported to ethics committees and regulatory authorities in accordance with regulations. Researchers periodically review all adverse events, and assess the risks and benefits of the study when necessary.

**8.6 Statistics**

This is an open label, exploratory development study to evaluate the safety and tolerability, and persistence and engraftment of autologous T cells engineered to express a chimeric antigen receptor targeting CD19. The subject population to be analyzed for primary and secondary endpoints will include all patients who were infused with CART-19 cells. A second population of patients will include all patients who do not receive CART-19 cells. Reasons for patients who do not receive cell infusions are likely to include 1) ineffective transduction of autologous T cells that is below the minimal acceptable dose of 1 x 10^7^ CART-19 dose; 2) rapid progression, clinical deterioration, and/or death between the time of enrollment and infusion; 3) subject withdrawal.

**8.7 Subject Population(s) for Analysis**

Descriptive statistics were used to describe the patients’ baseline characteristics and the temporal profiles of severe cytopenia. The univariate and multivariate Logistic Regression model was applied to assess whether several variables were contributing factors to PHT. Categorical variables were analyzed using the Chi-square test. Descriptive and survival analyses were performed using the Kaplan-Meier methodology. A log-rank test was utilized to compare OS and EFS between patient groups.

**8.8 Ethical Considerations**

This protocol and any amendments will be submitted to a properly constituted independent Institutional Review Board (IRB), in agreement with local legal prescriptions, for formal approval of the study conduct. The decision of the IRB concerning the conduct of the study will be made in writing to the investigator and a copy of this decision will be provided to the sponsor before commencement of this study.

**8.9 Confidentiality**

The investigator must ensure anonymity of the patients and patients must not be identified by names in any documents submitted to the funding sponsor. Signed informed consent forms and patient enrollment log must be kept strictly confidential to enable patient identification at the site. Information about study subjects will be kept confidential and managed. In the event that a subject revokes authorization to collect, the investigator, by regulation, retains the ability to use all information collected prior to the revocation of subject authorization. For subjects that have revoked authorization to collect, attempts should be made to obtain permission to collect at least vital status (i.e. that the subject is alive) at the end of their scheduled study period.

1. **Faculty**

| **personnel** | **Position/major** | **task** |
| --- | --- | --- |
| Xu Kai-lin | Director /hematology | Protocol design and supervisor |
| Cao Jiang | Director/oncology | Protocol design and supervisor |
| Chong Chen | Deputy director of the physician /hematology | Protocol design |
| Zhao Li-na | Deputy director of the physician /hematology | Clinical observation |
| Yao Yue | Deputy director of the physician /hematology | Clinical observation |
| Chen Hai | Attending /hematology | Subject screen |
| Wang Jiao-jiao | Attending /hematology | Clinical observation |
| Sun Zeng-tian | Attending /hematology | Clinical observation |
| Li Zhen-yu | Director /hematology | Subject screen |
| Zeng Ling-yu | Director /hematology | Subject screen |
| Wu Qing-yun | Deputy director of the physician /hematology | Subject screen |
| Wang Gang | Assistant research fellow/cell therapy | Cell preparation |
| Shi Ming | Assistant research fellow/cell therapy | CAR-T preparation supervisor |
| Jing Guang-jun | Assistant research fellow/cell therapy | CAR-T Cell preparation |
| Lu Qun-xian | associate research fell ow/Medical examiner | Tests |
| Wu Qing-yun | associate research fellow /hematology | CAR-T Cell preparation |
| Zhao Jing | Nurse supervisor/nursing | Nurse |

1. **References**

1. Attal M, Harousseau JL, Stoppa AM, Sotto JJ, Fuzibet JG, Rossi JF, et al. A prospective, randomized trial of autologous bone marrow transplantation and chemotherapy in multiple myeloma. Intergroupe Francais du Myelome. N Engl J Med. 1996 Jul 11;335(2):91-7.

2. Barlogie B, Jagannath S, Desikan KR, Mattox S, Vesole D, Siegel D, et al. Total therapy with tandem transplants for newly diagnosed multiple myeloma. Blood. 1999 Jan 1;93(1):55-65.

3. Richardson PG, Barlogie B, Berenson J, Singhal S, Jagannath S, Irwin D, et al. A phase 2 study of bortezomib in relapsed, refractory myeloma. N Engl J Med. 2003 Jun 26;348(26):2609-17.

4. Rajkumar SV, Blood E. Lenalidomide and venous thrombosis in multiple myeloma. N Engl J Med. 2006 May 11;354(19):2079-80.

5. Lokhorst HM, Plesner T, Laubach JP, Nahi H, Gimsing P, Hansson M, et al. Targeting CD38 with Daratumumab Monotherapy in Multiple Myeloma. N Engl J Med. 2015 Sep 24;373(13):1207-19.

6. Palumbo A, Chanan-Khan A, Weisel K, Nooka AK, Masszi T, Beksac M, et al. Daratumumab, Bortezomib, and Dexamethasone for Multiple Myeloma. N Engl J Med. 2016 Aug 25;375(8):754-66.

7. McCarthy PL, Owzar K, Hofmeister CC, Hurd DD, Hassoun H, Richardson PG, et al. Lenalidomide after stem-cell transplantation for multiple myeloma. N Engl J Med. 2012 May 10;366(19):1770-81.

8. Krishnan A, Pasquini MC, Logan B, Stadtmauer EA, Vesole DH, Alyea E, 3rd, et al. Autologous haemopoietic stem-cell transplantation followed by allogeneic or autologous haemopoietic stem-cell transplantation in patients with multiple myeloma (BMT CTN 0102): a phase 3 biological assignment trial. Lancet Oncol. 2011 Dec;12(13):1195-203.

9. Kumar SK, Dimopoulos MA, Kastritis E, Terpos E, Nahi H, Goldschmidt H, et al. Natural history of relapsed myeloma, refractory to immunomodulatory drugs and proteasome inhibitors: a multicenter IMWG study. Leukemia. 2017 Nov;31(11):2443-8.

10. Brown RD, Spencer A, Ho PJ, Kennedy N, Kabani K, Yang S, et al. Prognostically significant cytotoxic T cell clones are stimulated after thalidomide therapy in patients with multiple myeloma. Leuk Lymphoma. 2009 Nov;50(11):1860-4.

11. Tyler EM, Jungbluth AA, O'Reilly RJ, Koehne G. WT1-specific T-cell responses in high-risk multiple myeloma patients undergoing allogeneic T cell-depleted hematopoietic stem cell transplantation and donor lymphocyte infusions. Blood. 2013 Jan 10;121(2):308-17.

12. Peggs KS, Thomson K, Hart DP, Geary J, Morris EC, Yong K, et al. Dose-escalated donor lymphocyte infusions following reduced intensity transplantation: toxicity, chimerism, and disease responses. Blood. 2004 Feb 15;103(4):1548-56.

13. Sadelain M. T-cell engineering for cancer immunotherapy. Cancer J. 2009 Nov-Dec;15(6):451-5.

14. Sadelain M, Brentjens R, Riviere I. The basic principles of chimeric antigen receptor design. Cancer Discov. 2013 Apr;3(4):388-98.

15. Bhojwani D, Pui CH. Relapsed childhood acute lymphoblastic leukaemia. Lancet Oncol. 2013 May;14(6):e205-17.

16. Geyer MB, Brentjens RJ. Review: Current clinical applications of chimeric antigen receptor (CAR) modified T cells. Cytotherapy. 2016 Nov;18(11):1393-409.

17. Garfall AL, Maus MV, Hwang WT, Lacey SF, Mahnke YD, Melenhorst JJ, et al. Chimeric Antigen Receptor T Cells against CD19 for Multiple Myeloma. N Engl J Med. 2015 Sep 10;373(11):1040-7.

18. Ramos CA, Savoldo B, Torrano V, Ballard B, Zhang H, Dakhova O, et al. Clinical responses with T lymphocytes targeting malignancy-associated kappa light chains. J Clin Invest. 2016 Jul 1;126(7):2588-96.

19. Carpenter RO, Evbuomwan MO, Pittaluga S, Rose JJ, Raffeld M, Yang S, et al. B-cell maturation antigen is a promising target for adoptive T-cell therapy of multiple myeloma. Clin Cancer Res. 2013 Apr 15;19(8):2048-60.

20. Ali SA, Shi V, Maric I, Wang M, Stroncek DF, Rose JJ, et al. T cells expressing an anti-B-cell maturation antigen chimeric antigen receptor cause remissions of multiple myeloma. Blood. 2016 Sep 29;128(13):1688-700.

21. Yaccoby S. The phenotypic plasticity of myeloma plasma cells as expressed by dedifferentiation into an immature, resilient, and apoptosis-resistant phenotype. Clin Cancer Res. 2005 Nov 1;11(21):7599-606.

22. O'Connor BP, Raman VS, Erickson LD, Cook WJ, Weaver LK, Ahonen C, et al. BCMA is essential for the survival of long-lived bone marrow plasma cells. J Exp Med. 2004 Jan 5;199(1):91-8.

23. Chauhan D, Singh AV, Brahmandam M, Carrasco R, Bandi M, Hideshima T, et al. Functional interaction of plasmacytoid dendritic cells with multiple myeloma cells: a therapeutic target. Cancer Cell. 2009 Oct 6;16(4):309-23.

24. Ryan MC, Hering M, Peckham D, McDonagh CF, Brown L, Kim KM, et al. Antibody targeting of B-cell maturation antigen on malignant plasma cells. Mol Cancer Ther. 2007 Nov;6(11):3009-18.

25. Xu S, Lam KP. B-cell maturation protein, which binds the tumor necrosis factor family members BAFF and APRIL, is dispensable for humoral immune responses. Mol Cell Biol. 2001 Jun;21(12):4067-74.

26. Rajkumar SV, Dimopoulos MA, Palumbo A, Blade J, Merlini G, Mateos MV, et al. International Myeloma Working Group updated criteria for the diagnosis of multiple myeloma. Lancet Oncol. 2014 Nov;15(12):e538-48.

27. Palumbo A, Rajkumar SV, San Miguel JF, Larocca A, Niesvizky R, Morgan G, et al. International Myeloma Working Group consensus statement for the management, treatment, and supportive care of patients with myeloma not eligible for standard autologous stem-cell transplantation. J Clin Oncol. 2014 Feb 20;32(6):587-600.

28. van Dongen JJ, Lhermitte L, Bottcher S, Almeida J, van der Velden VH, Flores-Montero J, et al. EuroFlow antibody panels for standardized n-dimensional flow cytometric immunophenotyping of normal, reactive and malignant leukocytes. Leukemia. 2012 Sep;26(9):1908-75.

29. Common Terminology Criteria for Adverse Events (CTCAE) Version 5. Published: November 30. Lee DW, Gardner R, Porter DL, Louis CU, Ahmed N, Jensen M, et al. Current concepts in the diagnosis and management of cytokine release syndrome. Blood. 2014 Jul 10;124(2):188-95.

Appendix

Appendix 1

Revised International Myeloma Working Group diagnostic criteria for multiple myeloma and smouldering multiple myeloma

Definition of multiple myeloma
Clonal bone marrow plasma cells ≥10% or biopsy-proven bony or extramedullary plasmacytoma and any one or more of the following myeloma defining events:
• Myeloma defining events:
• Evidence of end organ damage that can be attributed to the underlying

plasma cell proliferative disorder, specifically:
• Hypercalcaemia: serum calcium >0.25 mmol/L (>1 mg/dL) higher than

theupper limit of normal or >2.75 mmol/L (>11 mg/dL)
• Renal insufficiency: creatinine clearance <40 mL per min† or serum

creatinine>177 μmol/L (>2 mg/dL)
• Anaemia: haemoglobin value of >20 g/L below the lower limit of

normal, or ahaemoglobin value <100 g/L
• Bone lesions: one or more osteolytic lesions on skeletal radiography,

CT, orPET-CT

• Any one or more of the following biomarkers of malignancy:
• Clonal bone marrow plasma cell percentage ≥60%
• Involved: uninvolved serum free light chain ratio ≥100
• >1 focal lesions on MRI studies

Definition of smouldering multiple myeloma
Both criteria must be met:
• Serum monoclonal protein (IgG or IgA) ≥30 g/L or urinary monoclonal

Protein ≥500 mg per 24 h and/or clonally bone marrow plasma cells 10–60%
• Absence of myeloma defining events or amyloidosis

Appendix 2

KARNOFSKY PERFORMANCE STATUS SCALE DEFINITIONS RATING (%) CRITERIA

| PERFORMANCE STATUS | SCORE |
| --- | --- |
| Normal no complaints; no evidence of disease. | 100 |
| Able to carry on normal activity; minor signs or symptoms of disease. | 90 |
| Normal activity with effort; some signs or symptoms of disease. | 80 |
| Cares for self; unable to carry on normal activity or to do active work. | 70 |
| Requires occasional assistance, but is able to care for most of his personal needs. | 60 |
| Requires considerable assistance and frequent medical care. | 50 |
| Disabled; requires special care and assistance. | 40 |
| Severely disabled; hospital admission is indicated although death not imminent. | 30 |
| Very sick; hospital admission necessary; active supportive treatment necessary. | 20 |
| Moribund; fatal processes progressing rapidly. | 10 |
| Dead | 0 |

Appendix 3

CAR-T proliferation

The proliferation of CAR-T cells was constantly monitored after adoptively infusion into patients. The PBMCs were harvested from patients periodically and the average copy number of CAR gene in patients was determined by quantitative real-time PCR (QPCR). Briefly, the genomic DNA was purified using a Genomic DNA Purification Kit (Thermo Fisher Scientific). The plasmid containing human albumin or CAR was used to establish standard curves. The albumin gene served as an internal control due to the presence of its sequence in two copies per genome.

Procedure

1．DNA preparation

Genomic DNA extraction kit was used to extract genomic DNA from cells.

2. Standard curve

(1) The concentration of plasmid template (pAlb and LTR) was first adjusted to 1 mg/mL, and then the number of copies of the sample was calculated according to the number of bases in the plasmid.

(2) The first point of the standard curve is 1 x 10^7^ copies, then diluted by 10 times gradient, and the last point is 103 copies to produce the standard curve.

(3) Gradient dilution and standard curve are made according to the way shown below.

Appendix 4

RESPONSE CRITERIA FOR MULTIPLE MYELOMA (Revised Uniform Response Criteria by the International Myeloma Working Group)

| Response Category | Response Criteria |
| --- | --- |
| CR, complete response | Negative immunofixation of serum and urine, disappearance of any soft tissue plasmacytomas, and <5% plasma cells in bonemarrow; in patients for whom only measurable disease is by serum FLC level, normal FLC ratio of 0.26 to 1.65 in addition to CRcriteria is required; two consecutive assessments are needed |
| sCR, stringent complete response | CR as defied plus normal FLC ratio and absence of clonal plasma cells by immunohistochemistry or two- to four-color flow cytometry; two consecutive assessments of laboratory parameters are needed |
| Immunophenotypic CR | sCR as defied plus absence of phenotypically aberrant plasma cells (clone) in bone marrow with minimum of 1 million total bone marrow cells analyzed by multi-parametric flow cytometry (with >four colors)Molecular C |
| Molecular CR | CR as defied plus negative allele-specific oligonucleotide polymerase chain reaction (sensitivity 10-5) |
| VGPR, very good partial response | Serum and urine M component detectable by immunofixation but not on electrophoresis or ≥90% reduction in serum M componentplus urine M component <100 mg/24 h; in patients for whom only measurable disease is by serum FLC level, >90% decrease indifference between involved and uninvolved FLC levels, in addition to VGPR criteria, is required; two consecutive assessments are needed |
| PR, partial response | ≥50% reduction of serum M-protein and reduction in 24-hour urinary M-protein by ≥90% or to <200 mg per 24 hIf the serum and urine M-protein are unmeasurable, a ≥50% decrease in the difference between involved and uninvolved FLC levelsis required in place of the M-protein criteria. If serum and urine M-protein are unmeasurable, and serum free light assay is also unmeasurable, ≥50% reduction in plasma cells is required in place of M-protein, provided baseline bone marrow plasma cell percentage was ≥30%.In addition, if present at baseline, a ≥50% reduction in the size of soft tissue plasmacytomas is also required. Two consecutive assessments are needed; no known evidence of progressive or new bone lesions if radiographic studies were performed. |
| MR, minimal response for relapsed refractory myeloma only | ≥25% but ≤49% reduction of serum M protein and reduction in 24-hour urine M protein by 50% to 89% In addition, if present at baseline, 25% to 49% reduction in size of soft tissue plasmacytomas is also required No increase in size or number of lytic bone lesions (development of compression fracture does not exclude response) |
| SD, stable disease | Not meeting criteria for CR, VGPR, PR or progressive disease; no known evidence of progressive or new bone lesions if radiographic studies were performed |
| PD, progressive disease | Increase of 25% from lowest response value in any of following: Serum M component with absolute increase ≥0.5 g/dL; serum M component increases ≥1 g/dL are sufficient to defiedrelapse if starting M component is ≥5 g/dl and/or; Urine M component (absolute increase must be ≥200 mg/24 h) and/or; Only in patients without measurable serum and urine M protein levels: difference between involved and uninvolved FLC levels (absolute increase must be >10 mg/dl); Only in patients without measurable serum and urine M protein levels and without measurable disease by FLC level, bone marrow plasma cell percentage (absolute percentage must be ≥10%) Development of new or definite increase in size of existing bone lesions or soft tissue plasmas Development of hypercalcemia that can be attributed solely to |

Appendix 5

RESPONSE CRITERIA FOR MULTIPLE MYELOMA（RELAPSED）

| Relapse Subcategory | Relapse Criteria |
| --- | --- |
| Clinical relapse | Clinical relapse requires one or more of: •Direct indicators of increasing disease and/or end organ dysfunction (CRAB features). It is not used in calculation of time to progression or progression-free survival but is listed here as something that can be reported optionally or for use in clinical practice • Development of new soft tissue plasmacytomas or bone lesions • Definite increase in the size of existing plasmacytomas or bone lesions. A definite increase is defined as a 50% (and at least 1 cm) increase as measured serially by the sum of the products of the cross-diameters of the measurable lesion • Hypercalcemia (>11.5 mg/dL) [2.65 mmol/L] • Decrease in hemoglobin of ≥2 g/dL [1.25 mmol/L] • Rise in serum creatinine by 2 mg/dL or more [177 µmol/L or more] |
| Relapse from CR | Any one or more of the following: • Reappearance of serum or urine M-protein by immunofixation or electrophoresis • Development of ≥5% plasma cells in the bone marrow • Appearance of any other sign of progression (ie, new plasmacytoma, lytic bone lesion, or hypercalcemia) |

Appendix 6

MRD

Bone marrow was collected at different follow-up time after treatment. MRD was detected according to EuroFlow antibody panels for plasma cell disorders (PCD). In order to clarify the expression of BCMA in different patients before treatment, we added anti-BCMA antibody on the basis of panel. Multiple myeloma Flow-MRD: antibody panel

| Tube | Qdot 565 | APC-CY7 | APC | PerCP-CY5.5 | PE-CY7 | BV510 | V450 | FITC | PE |
| --- | --- | --- | --- | --- | --- | --- | --- | --- | --- |
| 1 | CD45 | CD38 | CD138 | CD19 | BCMA | CD27 | CD56 |  |  |
| 2 | CD45 | CD38 | CD138 | CD19 | BCMA |  |  | CyIgκ | CyIgλ |

Appendix 7

CRS Toxicity Grading

| Grade | Criteria |
| --- | --- |
| 1 | Mild reaction:  Treated with supportive care such as antipyretics, antiemetics. |
| 2 | Moderate reaction requiring IV therapies or parenteral nutrition; some signs organ dysfunction (i.e. grade 2 creatinine or grade 3 liver function tests) related to CRS and not attributable to any other condition. Hospitalization for management of CRS related symptoms including fevers associated neutropenia |
| 3 | More severe reaction: Hospitalization required for management of symptoms related to organ dysfunction including grade 4 LFTs or grade 3 creatinine related to CRS and not attributable to any other conditions; this excludes management of fevers or myalgias. Includes hypotension treated with IVFs or low-dose pressors, coagulopathy requiring fresh frozen plasma (FFP) or cryoprecipitate, and hypoxia requiring supplemental oxygen (nasal cannula oxygen, high flow oxygen, Continuous Positive Airway Pressure [CPAP] or Bilateral Positive Airway Pressure [BiPAP]). Patients admitted for management of suspected infection due to fevers and/or neutropenia may have grade 2 CRS. |
| 4 | Life-threatening complications such as hypotension requiring pressors, hypoxia requiring mechanical ventilation. |
| 5 | Death |

Appendix 8

STAGING SYSTEMS FOR MULTIPLE MYELOMA

| Stage | Durie-Salmon Criteria |
| --- | --- |
| Ⅰ | All of the following: • Hemoglobin value >10 g/dL • Serum calcium value normal or ≤12 mg/dL • Bone x-ray, normal bone structure) orsolitary bone  plasmacytoma only • Low M-component production rate： IgG value <5 g/dl; IgA value <3 g/dl Bence Jones protein <4 g/24 h |
| Ⅱ | Neither stage l nor stage lll |
| Ⅲ | One or more of the following: • Hemoglobin value <8.5 g/dl • Serum calcium value >12 mg/dl • Advanced lytic bone lesions • High M-component production rate： IgG value >7 g/dl; IgA value >5 g/dl Bence Jones protein >12 g/24 h |
| Subclassifiation Criteria A Normal renal function (serum creatinine level <2.0 mg/dl) B Abnormal renal function (serum creatinine level ≥2.0 mg/dl) | |

Appendix 9

|  |  | CRS clinical observation | |  | date： | |  |  |
| --- | --- | --- | --- | --- | --- | --- | --- | --- |
| observation | 8:00 | 10:00 | 12:00 | 14:00 |  | 16:00 | 18:00 |  |
| fever |  |  |  |  |  |  |  |  |
| chilling |  |  |  |  |  |  |  |  |
| fatigue |  |  |  |  |  |  |  |  |
| anorexia |  |  |  |  |  |  |  |  |
| Muscle pain |  |  |  |  |  |  |  |  |
| Joint pain |  |  |  |  |  |  |  |  |
| Nosia/vomitting |  |  |  |  |  |  |  |  |
| diarrhea |  |  |  |  |  |  |  |  |
| rush |  |  |  |  |  |  |  |  |
| pant |  |  |  |  |  |  |  |  |
| palpitation |  |  |  |  |  |  |  |  |
| headache |  |  |  |  |  |  |  |  |
| Psycho status |  |  |  |  |  |  |  |  |
| consciousness |  |  |  |  |  |  |  |  |
| Delirium |  |  |  |  |  |  |  |  |
| aphasia |  |  |  |  |  |  |  |  |
| illusion |  |  |  |  |  |  |  |
| Tremor |  |  |  |  |  |  |  |
| epilepsy |  |  |  |  |  |  |  |
| heart rate |  |  |  |  |  |  |  |
| blood pressure |  |  |  |  |  |  |  |
| Oxygen saturation |  |  |  |  | |  |  |

Appendix 10

SOP of Serious Adverse Reactions during CART Treatment

Sepsis

1. Diagnosis

2010 Diagnostic criteria for sepsis, severe sepsis and septic shock in Germany


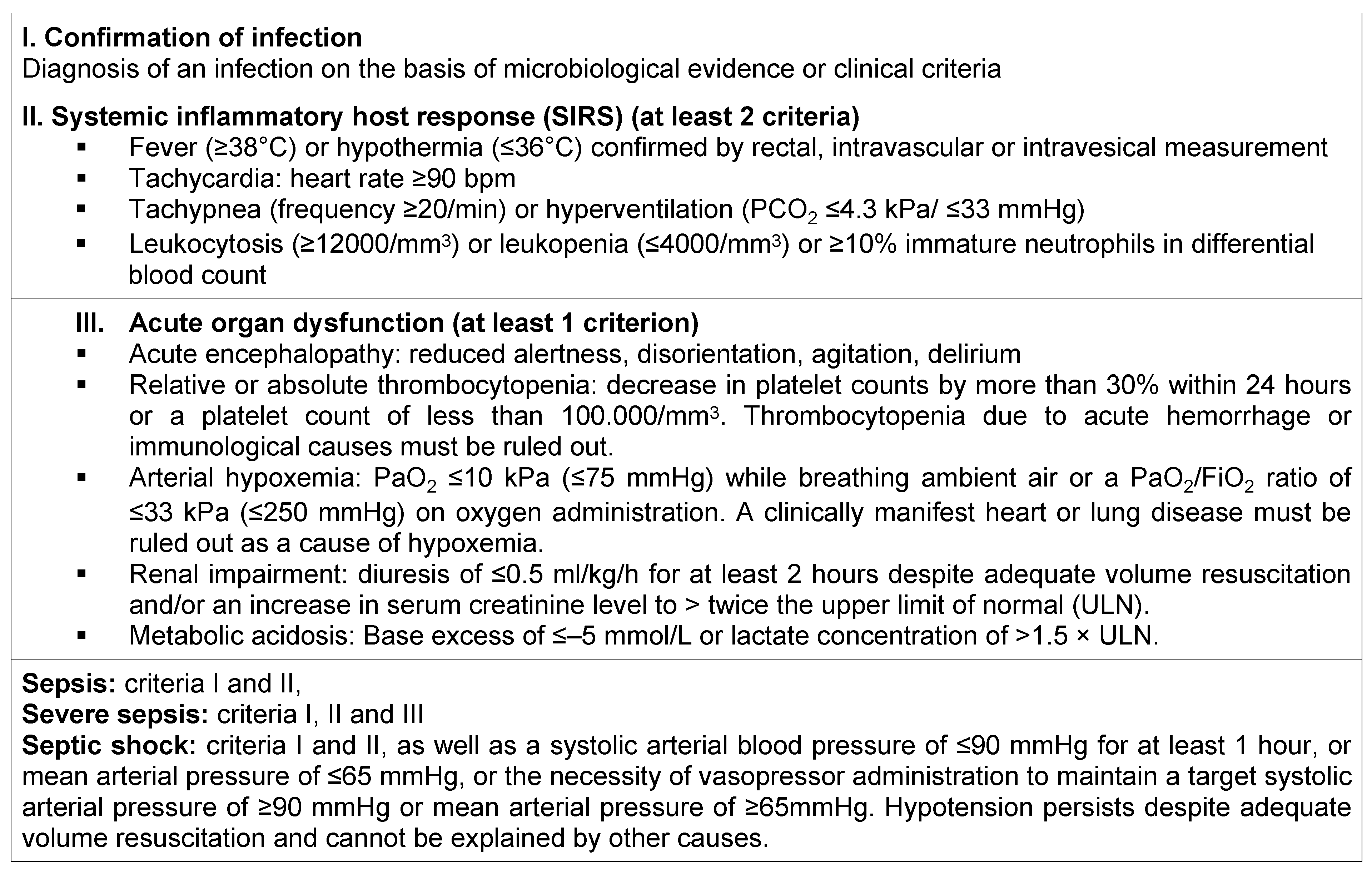


2012 International Diagnostic Criteria for Sepsis

2012 International Diagnostic Criteria for Severe Sepsis

### Therapy

A. Initial resuscitation

We recommend the protocolized, quantitative resuscitation of patients with sepsis-induced tissue hypoperfusion (defined in this document as hypotension persisting after initial fluid challenge or blood lactate concentration ≥ 4 mmol/L. This protocol should be initiated as soon as hypoperfusion is recognized and should not be delayed pending ICU admission. During the first 6 h, the goals of initial resuscitation of sepsis-induced hypoperfusion should include all of the following as a part of a treatment protocol:

(a) CVP 8-12 mmHg

(b) MAP C65 mmHg

(c) Urine output ≥0.5 mL kg/ h^-1^

(d) Superior vena cava oxygenation saturation (ScvO2) or mixed venous oxygen saturation (SvO2) 70 or 65 %, respectively.

2、During the first 6 h of resuscitation, if ScvO2 less than 70 %or SvO2 equivalent of less than 65 % persists with what is judged to be adequate intravascular volume repletion in the presence of persisting tissue hypoperfusion, then dobutamine infusion (to a maximum of 20 ug/kg. min) or transfusion of packed red blood cells to achieve a hematocrit of greater than or equal to 30 % in attempts to achieve the ScvO2 or SvO2 goal are options.

3、In patients with elevated lactate levels targeting resuscitation to normalize lactate as rapidly as possible.

B. Diagnosis

1、Cultures as clinically appropriate before antimicrobial therapy if no significant delay (45 min) in the start of antimicrobial. At least 2 sets of blood cultures (both aerobic and anaerobic bottles) be obtained before antimicrobial therapy with at least 1 drawn percutaneously and 1 drawn through each vascular access device, unless the device was recently (<48 h) inserted. Other culture specimens, including urine, cerebrospinal fluid, wounds, respiratory secretions or other body fluids that may be the source of infection, should be retained before antibiotics are used.

2、Imaging studies performed promptly to confirm a potential source of infection.

3、Use of the 1,3 b-D-glucan assay (grade 2B), mannan and anti-mannan antibody assays , if available and invasive candidiasis is in differential diagnosis of cause of infection.

C. Antimicrobial therapy

1、Administration of effective intravenous antimicrobials within the first hour of recognition of septic shock (grade 1B) and severe sepsis without septic shock as the goal of therapy.

2、Initial empiric anti-infective therapy of one or more drugs that have activity against all likely pathogens (bacterial and/or fungal or viral) and that penetrate in adequate concentrations into tissues presumed to be the source of sepsis.

3、Duration of therapy typically 7–10 days; longer courses may be appropriate in patients who have a slow clinical response, undrainable foci of infection, bacteremia with S. aureus; some fungal and viral infections or immunologic deficiencies, including neutropenia.

４、If the patient's current clinical symptoms are determined to be caused by non-infectious factors, antibiotic treatment should be discontinued immediately.

5、Procalcitonin is not recommended as a diagnostic marker for severe sepsis. During antibiotic treatment, if no infection is found, clinicians are advised to use low procalcitonin levels as a marker for stopping empirical antibiotic treatment.

D. Infection prevention

1a、For some specific infections requiring urgent treatment, such as necrotizing fasciitis, diffuse peritonitis, cholangitis, intestinal infarction, the etiology should be identified and diagnosed as soon as possible, and the diagnosis should be completed within 6 hours of symptom onset.

1b、Determine whether there are controllable sources of infection. Control measures include drainage of abscess or local infection, debridement of necrotic tissue after infection, removal of medical devices that can cause infection, or control of the source of microbial infection.

2、when infected peripancreatic necrosis is identified as a potential source of infection, definitive intervention is best delayed until adequate demarcation of viable and nonviable tissues has occurred .

3、When source control in a severely septic patient is required, the effective intervention associated with the least physiologic insult should be used (e.g., percutaneous rather than surgical drainage of an abscess.

4、If intravascular access devices are a possible source of severe sepsis or septic shock, they should be removed promptly after other vascular access has been established

(UG).

E. Fluid therapy of severe sepsis

1、We recommend crystalloids be used as the initial fluid of choice in the resuscitation of severe sepsis and septic shock.

2、The initial goal of fluid resuscitation is to achieve a CVP of at least 8 mmHg (12 mmHg for mechanical ventilation patients), and further fluid therapy is usually required.

3a、We recommend that a fluid challenge technique be applied wherein fluid administration is continued as long as there is hemodynamic improvement either based on dynamic (e.g., change in pulse pressure, stroke volume variation) or static (e.g., arterial pressure, heart rate) variables.

3b、An initial fluid challenge in patients with sepsis-induced tissue hypoperfusion with suspicion of hypovolemia to achieve a minimum of 30 mL/ kg of crystalloids (a portion of this may be albumin equivalent). More rapid administration and greater amounts of fluid may be needed in some patients

F. Vasopressors

1、recommend that vasopressor therapy initially target MAP of ≥65mmHg.

2、norepinephrine as the first-choice vasopressor .epinephrine (added to and potentially substituted for norepinephrine) when an additional agent is needed to maintain adequate blood pressure. Vasopressin (up to 0.03 U/min) can be added to norepinephrine with the intent of raising MAP to target or decreasing norepinephrine dosage.

3、Use of low-dose dopamine as a renal protective drug is not recommended。

4、All patients requiring vasopressors have an arterial catheter placed as soon as practical if resources are available

G. Inotropic therapy

1、In patients with myocardial dysfunction (elevated filling pressure and decreased cardiac output) or persistent insufficiency of perfusion, intravenous dobutamine or vasopressin are administered even if the blood volume is adequate and the mean arterial pressure is greater than or equal to 65 mmHg.

2、We recommend against the use of a strategy to increase cardiac index to predetermined supranormal levels.

H. Corticosteroids

1、We suggest not using intravenous hydrocortisone as a treatment of adult septic shock patients if adequate fluid resuscitation and vasopressor therapy are able to restore hemodynamic stability (see goals for Initial Resuscitation). If this is not achievable, we suggest intravenous hydrocortisone alone at a dose of 200 mg per day

2、We suggest not using the ACTH stimulation test to identify the subset of adults with septic shock who should receive hydrocortisone（2B）。

3、If hydrocortisone cannot be obtained, and the replacement hormone preparation has no significant corticosteroid activity, it is recommended to increase the daily oral fluorocortisone (50 ug). If hydrocortisone is used, fluorocortisone is optional.

4、We suggest that clinicians taper the treated patient from steroid therapy when vasopressors are no longer required.

5、It is recommended that the daily dose of glucocorticoid in severe sepsis or septic shock patients should not exceed 300 mg equivalent of hydrocortisone.

7、We recommend that corticosteroids not be administered for the treatment of sepsis in the absence of shock. When low-dose hydrocortisone is given, we suggest using continuous infusion rather than repetitive bolus injections.

I. Blood product administration

1、Once tissue hypoperfusion has resolved and in the absence of extenuating circumstances, such as myocardial ischemia, severe hypoxemia, acute hemorrhage, or ischemic coronary artery disease, we recommend that red blood cell transfusion occur when the hemoglobin concentration decreases to 7.0 g/dL to target a hemoglobin concentration of 7.0–9.0 g/dL in adults.

2、 We recommend not using erythropoietin as a specific treatment of anemia associated with severe sepsis.

3、 We suggest that fresh frozen plasma not be used to correct laboratory clotting abnormalities in the absence of bleeding or planned invasive procedures

4、 We recommend against antithrombin administration for the treatment of severe sepsis and septic shock

5、In patients with severe sepsis, we suggest that platelets be administered prophylactically when counts are <10,000/mm3 (10 ×10^9^/L) in the absence of apparent

bleeding, as well when counts are B20,000/mm3 (20 ×10^9^/L) if the patient has a significant risk of bleeding. Higher platelet counts ≥50,000/mm3(50 ×10^9^/L) are advised for active bleeding, surgery, or invasive procedures.

Tumor lysis syndrome

### Diagnosis

| Laboratory tumor lysis syndrome（LTLS）：2 or more |
| --- |
| - uric acid > 8 mg/dL or 25% increase - potassium > 6 meq/L or 25% increase - phosphate > 4.5 mg/dL or 25% increase - calcium < 7 mg/dL or 25% decrease |
| note：abnormality in two or more of the following, occurring within three days before or seven days after chemotherapy. |
| Clinical tumor lysis syndrome（CTLS）：It meets the diagnostic criteria of LTLS, and one or more criteria can be diagnosed. |
| - increased serum creatinine (1.5 times upper limit of normal) - cardiac arrhythmia or sudden death - seizure |
| note：  1. Exclude the above changes caused by side effects of drugs;  2. Definition of normal upper limit of serum creatinine (ULN): 61.6 micromol/L for patients aged 1 to 12, 88 micromol/L for patients aged 12 to 16, 114.4 micromol/L for males aged 16 and 105.6 micromol/L for females. |

Cairo-Bishop Grading Classification of TLS


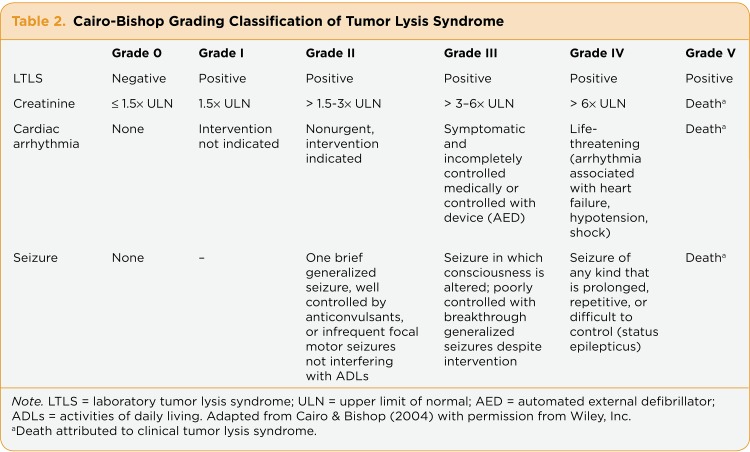


### Prevention of TLS


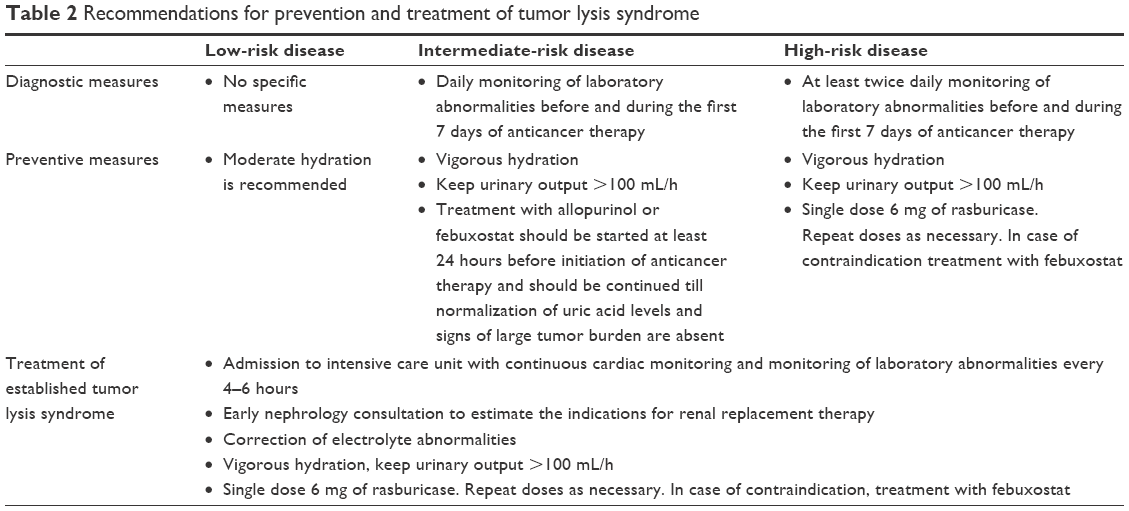


1. Therapy

3.1Intravenous hydration and Urinary alkalinization

Aggressive intravenous hydration at a rate of 2 to 3 L/day, maintaining a urine output of 100 to 200 mL/ hour. Mannitol 0.5 mg/kg or furosemide 0.5-1.0 mg/kg can be used as urine agent. The dosage was 2-4 mg/kg when hypouria or anuria occures, and the relative density of urine was 1.010-1.015.

Treating Electrolyte disorders

1. Hyperphosphatemia

Phosphate concentration ≥2.1mmol/L(children) or ≥1.45mmol/L(adult)。

Phosphate should be avoided. Aluminum hydroxide can be administered 15 mL/time and orally 50-100 mg/(kg.d) four times a day. Severe illness: dialysis, continuous arteriovenous hemofiltration and continuous venovenous hemofiltration (CVVH) can be used.

1. Hypocalcemia:≤1.75mmol /L.

Asymptomatic patients: no treatment for the time being;

Symptomatic patients: intravenous injection of calcium gluconate 50-100 mg/kg.

1. Hyperkalemia:≥6.0mmol/L.

Asymptomatic patients: oral or intravenous potassium supplementation should be avoided; ECG or ECG monitoring; sodium polystyrene sulfonate can be given (1 g/kg oral or enema).

Severe illness: When serum potassium is more than 7.0 mmol/L, in addition to the above methods, 10% calcium gluconate 100-200 mg/kg intravenous injection, 25% glucose (2mL/kg) +insulin 0.1U/kg intravenous drip, or even hemodialysis can be used.

1. hyperuricemia：Uric acid≥476mol/L，occurs 48～72 h post chemotherapy

Allopurinol:

Adults were given 100 mg/m^2^ or 10 mg/(kg.d) orally three times daily (maximum dose 800 mg/d) or 200-400 mg/(m^2^.d) or intravenously injected 3 times daily (maximum dose 600 mg/d).

300-450 mg/(m^2^.d) was given orally three times daily to children, the maximum dose was 400 mg/(m^2^.d).

Reduced dose in patients with renal failure，200 mg/d when creatinine clearance is 0.33-0.17 mL/s；100 mg/d when creatinine clearance is less than 0.17 mL/s.

When allopurinol is applied, the dosage of 6-MP or MP should be reduced to 65%-75%.

Indicators of Allopurinol in the Treatment of Hyperuricemia:

A. Normal uric acid;

B. The types of tumors were Hodgkin's lymphoma and chronic myeloid leukemia without blood invasion.

C. Tumor load is small, such as white blood cell count (WBC) less than 50 *109/L and lactate dehydrogenase (LDH) less than 2 times normal.

D. Chemotherapy intensity is low.

E. Tumors have no renal infiltration.

3.4 Treatment of renal failure (uremia)

Monitor fluid intake, electrolyte and blood pressure. Treatment of hyperuricemia and hyperphosphatism, prevention and treatment of uric acid nephropathy, adjust the dose of renal excretion drugs.If necessary, dialysis (blood-peritoneum) or blood purification (CAVH, CVVH) were performed.

Clinical signs and symptoms associated with CRS

| Organ system | Symptoms |
| --- | --- |
| Constitutional | Fever ± rigors, malaise, fatigue, anorexia, myalgias, arthalgias, nausea, vomiting, headache |
| Skin | Rash |
| Gastrointestinal | Nausea, vomiting, diarrhea |
| Respiratory | Tachypnea, hypoxemia |
| Cardiovascular | Tachycardia, widened pulse pressure, hypotension, increased cardiac output (early), potentially diminished cardiac output (late) |
| Coagulation | Elevated D-dimer, hypofibrinogenemia ± bleeding |
| Renal | Azotemia |
| Hepatic | Transaminitis, hyperbilirubinemia |
| Neurologic | Headache, mental status changes, confusion, delirium, word finding difficulty or frank aphasia, hallucinations, tremor, dymetria, altered gait, seizures |

1. Grading

| Classification | toxicity |
| --- | --- |
| Grade 1 | Mild reaction, infusion interruption not indicated; intervention not indicated |
| Grade 2 | Therapy or infusion interruption indicated but responds promptly to symptomatic treatment (e.g., antihistamines, NSAIDS, narcotics, IV fluids); prophylactic medications indicated for <=24 hrs |
| Grade 3 | Prolonged (e.g., not rapidly responsive to symptomatic medication and/or brief interruption of infusion); recurrence of symptoms following initial improvement; hospitalization indicated for clinical sequelae (e.g., renal impairment, pulmonary infiltrates) |
| Grade 4 | Life-threatening consequences; pressor or ventilatory support indicated |
| Grade 5 | dead |

therapy

| Grading | Therapy |
| --- | --- |
| Ⅰ | 1. support therapy  (including empirical anti-infective therapy, maintaining fluid balance and blood pressure)  2. Infection assessment  （(If fever and neutropenia occur, symptomatic treatment；Monitor fluid balance and use antipyretics and analgesics when necessary） |
| Ⅱ | 1. Non-elderly patients or no extensive complications, giving supportive therapy 2. Elderly patients or extensive complications, refer to the level III treatment plan 3. Close monitoring of heart function |
| Ⅲ/Ⅳ | 1. Actively support therapy, recommend 1-to-1 nursing, closely monitor cardiac function. 2. Tassimab(Initial dose is 4 mg/kg for adults and 8 mg/kg for children. If there is no clinical improvement, it can be reused within 24-48 hours) 3. Corticosteroids(Dexamethasone 0.5 mg/kg, maximum dosage 10 mg, is recommended for patients with severe neurological symptoms who are initially treated with methylprednisolone 2 mg/kg/d. |

- B cell deficiency
- Clinical indications of intravenous immunoglobulin infusion

①IgG< 2g/L;

②IgG 2-5g/L，Antibody defect and frequently bacterial infection.

③IgG 2-5g/L，Antibody defect and severe bacterial infection.

Dose of intravenous immunoglobulin

The initial dose was 0.4g-0.6g/kg/month.

To monitor IgG valley concentration (after the end of a diagnosis and treatment cycle), ensure that IgG valley concentration should be close to or higher than the lower limit of IgG valley concentration in normal people.
